# Multidimensional Physiological and Gut Microbiome Profiling Identifies Subtle Physiological Patterns in an Apparently Healthy Aging Indian Cohort: A Cross-Sectional Observational Study

**DOI:** 10.3390/microorganisms14092097

**Published:** 2026-09-19

**Authors:** Gangaraju Divyashri, Harini Hutti, Lalitha Prasanna Madduri Venkata, Bipin Pradeep Kumar, Ayako Yachie, Samik Ghosh

**Affiliations:** 1 Bioproducts Research, Iom Bioworks Pvt. Ltd., Centre for Cellular and Molecular Platforms (C-CAMP), GKVK Campus, Bengaluru 560 065, India; harini@iombio.com (H.H.); lalitha@iombio.com (L.P.M.V.); bipin@iombio.com (B.P.K.); 2 SBX Biosciences Inc., 885 West Georgia Street, 20th Floor, Vancouver, BC V6C 3E8, Canada; yachie@sbi.jp; 3 The Systems Biology Institute, Tokyo 141-0022, Japan

**Keywords:** apparently healthy aging, composite domain scores, multidimensional physiological health index (MPHI), gut microbiota, metabolic and inflammatory markers, psychophysiological health

## Abstract

Aging is characterized by gradual shifts in cellular and physiological processes, even in individuals who remain apparently clinically healthy. These changes reflect a coordinated decline in metabolic efficiency, immune regulation, organ function, psychological resilience, and sleep integrity, domains increasingly recognized as interconnected determinants of apparently healthy aging. This study explored age- and sex-associated patterns in multidimensional physiological domains and gut microbiota features among 45 apparently healthy middle-aged and elderly participants across four age–sex groups (middle-aged males, *n* = 13; middle-aged females, *n* = 11; elderly males, *n* = 13; elderly females, *n* = 8). Comprehensive clinical, metabolic, inflammatory, hepato-renal, psychological, and sleep parameters were standardized into domain-specific Z-scores, which were integrated into a composite multidimensional physiological health index (MPHI), a systems-level physiological index developed as a proxy for physiological processes associated with mitochondrial function and apparently healthy aging rather than a direct measure of mitochondrial biology. Whole-metagenome sequencing and MetaPhlAn-based taxonomic profiling were used to characterize gut microbiota composition and diversity. Despite no statistically significant overall differences among the four age–sex groups in multivariate analysis (MANOVA, *p* = 0.103) and gut microbial community structure (PERMANOVA, *p* = 0.651), descriptive variation was observed across several physiological domains, while microbiota–host associations remained exploratory and nominal after FDR correction. Middle-aged males showed comparatively less favorable metabolic and inflammatory profiles, while selected psychosocial and sleep-related measures showed variation across the age–sex groups, particularly among elderly females. Principal component analysis (PCA) of domain scores indicated multidimensional physiological variation influenced by inflammatory, metabolic, and hepato-renal indices, although substantial overlap was observed among the age–sex groups. The MPHI showed descriptive variation across the groups, with elderly males showing the highest composite scores and middle-aged females the lowest. Overall, the apparently healthy cohort showed subtle variation across interconnected physiological domains in the absence of overt differences in gut microbial community structure. These findings support the potential utility of composite physiological indices for exploratory characterization of multidimensional physiological variation during aging, while highlighting the need for validation in larger independent cohorts and against direct mitochondrial biomarkers.

## 1. Introduction

Aging is a complicated, multifaceted process that increases vulnerability to a variety of health issues [1]. A major scientific challenge is to understand the mechanisms that drive this gradual decline so that its negative effects can be reduced. Age consistently emerges as the primary risk factor for most chronic diseases, and growing evidence links early signs of aging to the accumulation of molecular stress and damage within cells [2]. Furthermore, these cellular abnormalities gradually change tissue homeostasis, inflammatory control, and metabolic balance, establishing the biological milieu in which age-related illnesses eventually arise [3]. Therefore, studying apparently healthy individuals has become vital for identifying the underlying biological mechanisms associated with apparently healthy aging because many of these early changes occur before clinical symptoms manifest. This perspective has broadened the focus of aging research beyond disease states, emphasizing the need to understand how molecular, metabolic, and physiological markers change naturally across the lifespan [4]. Among the many cellular systems affected during this early phase of aging, mitochondrial function has emerged as an important component of the biological processes underlying age-related physiological changes [5].

Against this backdrop, mitochondria have become central to discussions of how aging unfolds. Their deterioration has been a long-standing focus in aging biology, with the mitochondrial theory of aging proposing that impaired cellular respiration and increased production of reactive oxygen species contribute to age-related decline [6]. Beyond supplying cellular energy, mitochondria are essential for regulating calcium homeostasis [7], synthesizing heme [8], controlling cell growth [9], and coordinating programmed cell death [10]. Because mitochondria participate in such a diverse range of cellular processes, disruptions to their function can influence numerous molecular and physiological pathways. Declining mitochondrial function has been proposed to contribute to dysregulation across these interconnected systems, potentially contributing to age-related changes across tissues.

Recent research has increasingly underscored the close interplay between aging, gut microbial dynamics, and mitochondrial function [11,12,13,14]. The complex microbial ecosystem found in the human gut varies in composition and metabolic output as we age and these age-related shifts have been associated with multiple physiological domains, including metabolic regulation, inflammatory activity, hepato-renal homeostasis, psychological well-being, and sleep quality [15]. Increasing evidence supports the gut–brain axis as a bidirectional communication network through which gut microbial composition and metabolites can influence neuroimmune signaling, neurotransmitter pathways, stress responses, mood and sleep, while central nervous system signals can in turn modulate gastrointestinal physiology and microbial activity [16,17]. Changes in microbial metabolites and community structure have been associated with alterations in immune signaling, modulate systemic inflammation, nutrient processing, and metabolic homeostasis, highlighting that the gut microbiome is a key contributor to several interconnected aspects of biological aging [12]. Furthermore, a significant portion of the available literature is derived from clinically unwell or heterogeneous populations, which makes it challenging to differentiate between biological changes brought on by aging and changes caused by illness [18,19,20]. Consequently, multidimensional physiological variation and its relationship with gut microbiome features across age and sex-defined groups within apparently healthy aging populations remain insufficiently characterized.

To address this gap, the present WHO-registered clinical study focused exclusively on apparently healthy individuals across two age groups, middle-aged and elderly, and included both male and female participants to characterize age- and sex-specific patterns of apparently healthy aging. Specifically, the study explored whether multidimensional physiological profiles and gut microbiome features vary across age- and sex-defined groups within an apparently healthy cohort. The study combined gut microbiota characterization with a multidimensional panel of blood-based biomarkers and validated measures of psychological well-being and sleep quality. The biomarker panel incorporated metabolic indicators, inflammatory markers, hepato-renal parameters, and measures related to psychological well-being and sleep quality, allowing for an integrated assessment of multiple dimensions of physiological aging. These domains were subsequently integrated into a composite physiological index (MPHI) to provide a multidimensional assessment of physiological health across metabolic, inflammatory, hepato-renal, psychological well-being, and sleep-related domains. Parallel analysis of gut microbial composition offered an opportunity to examine how differences in microbial diversity and taxonomic composition correspond with these biomarker patterns. We hypothesized that aging would be associated with differences in multidimensional physiological profiles and gut microbiome diversity and composition and that specific gut microbiota features would show exploratory associations with host physiological measures. This study aimed to explore relationships among systemic physiological measures and gut microbiome characteristics across age- and sex-defined groups within an apparently healthy cohort. By integrating host physiological measures with gut microbiome features, the study sought to characterize multidimensional physiological and microbiome signatures that can inform the monitoring and support of apparently healthy aging.

## 2. Materials and Methods

### 2.1. Ethical Clearance Declaration

This study received approval from the Institutional Ethics Committee of the Trans-Disciplinary University (TDU), Bangalore, and was carried out in alignment with the ethical principles outlined in the Declaration of Helsinki. The committee reviewed and approved the study protocol TDU/IEC/2023/3 on 3 July 2023. All participants provided written informed consent after being informed about the study’s purpose, procedures, and any foreseeable risks. The work adhered to all applicable national ethical regulations.

### 2.2. Study Design and Participants

This exploratory clinical study examined apparently healthy aging by assessing systemic biochemical markers alongside gut microbiome composition in a middle-aged and elderly population. The study followed an observational, cross-sectional design, focusing on how metabolic, inflammatory, hepato-renal, psychological, and sleep-related indicators relate to gut microbial features across age and sex categories. The protocol was registered with the Clinical Trials Registry—India (CTRI/2023/07/055467, date 21 July 2023).

Participants were recruited from Bangalore through collaboration among the Trans-Disciplinary University (TDU), I-AIM Hospital, and Iom Bioworks Pvt. Ltd. Apparently healthy community-dwelling adults aged 40–80 years were invited to participate. Eligibility required that individuals had no self-reported history of major chronic diseases, including diabetes mellitus and hypertension. Screening included assessment of blood pressure and fasting blood glucose to identify potential abnormalities relevant to eligibility. Participants were also screened for acute illness, gastrointestinal disorders, and recent use of medications known to influence the gut microbiota, including antibiotics and probiotics within the previous six weeks. Information on potentially relevant lifestyle and demographic factors, including dietary intake, physical activity, smoking status, alcohol consumption, socioeconomic status, and pregnancy/lactation status, was collected using a structured study questionnaire. These variables were recorded for participant characterization but were not systematically incorporated as covariates in the present microbiome and physiological analyses. Eligibility was determined based on participant-reported medical history and the study screening assessments; medical records, previous laboratory reports, or formal clinical examinations were not used as additional eligibility criteria.

**Figure 1:**
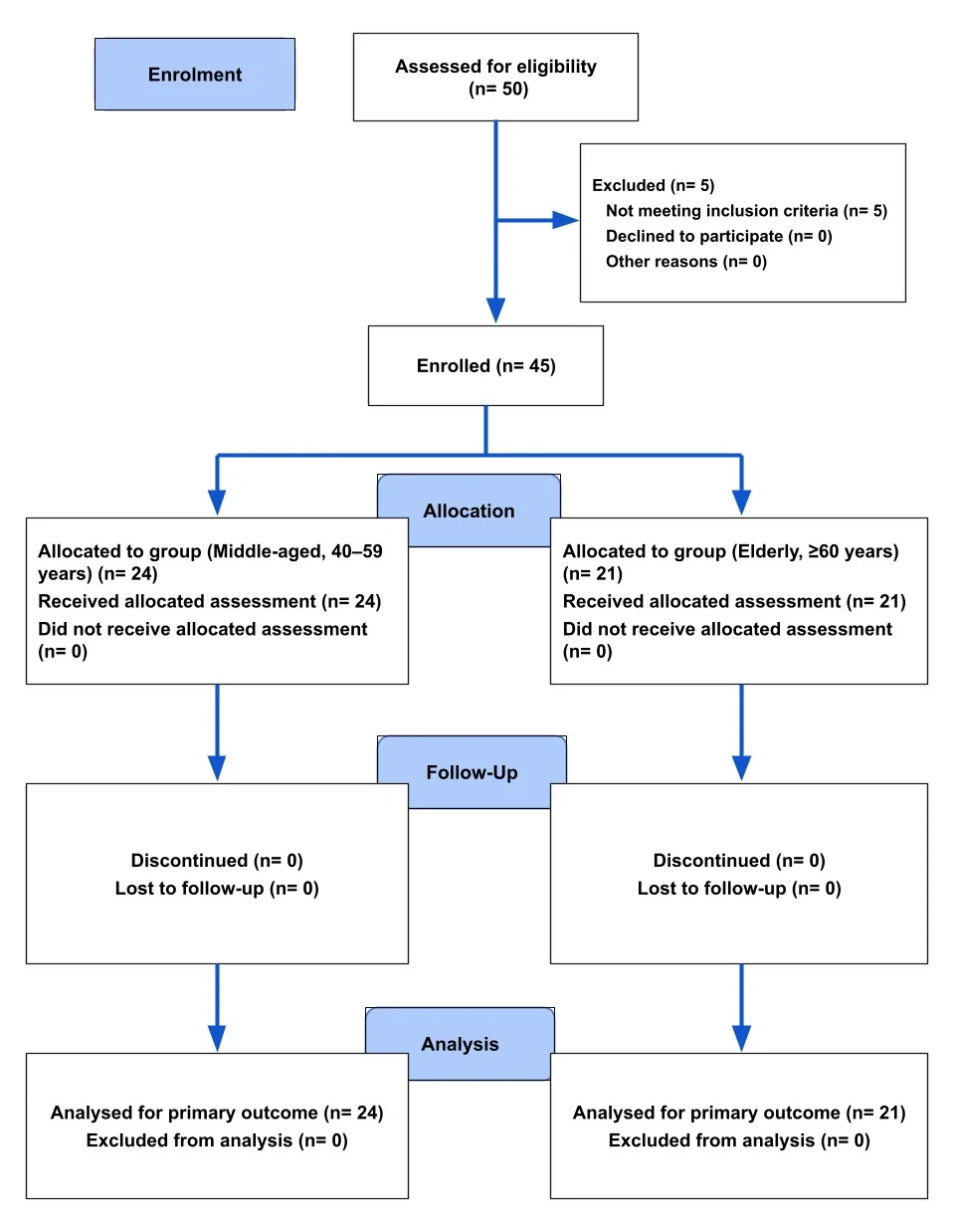
Participant flow diagram illustrating participant recruitment, eligibility assessment, enrolment, and inclusion in the physiological and gut microbiome analyses.

Participants (A total of 45 participants) meeting these criteria were enrolled and categorized into middle-aged and elderly groups for comparative analysis, with further stratification by sex (Figure 1). The dataset included a comprehensive panel of blood-based parameters covering metabolic, inflammatory, and hepato-renal indices along with validated questionnaires assessing psychological well-being and sleep quality. Stool samples were used to profile gut microbial composition. Together, these measures created a multidimensional dataset enabling exploration of how host physiology and microbiome characteristics vary across age groups within an apparently healthy aging cohort.

### 2.3. Sample Collection and DNA Extraction

Participants collected fresh stool samples at home using a provided kit containing a DNA stabilization buffer, which allowed microbial DNA to remain intact at room temperature for up to 30 days. Samples were maintained in the stabilization buffer at room temperature and returned to the study team within 72 h of collection. Upon receipt, samples underwent initial quality checks at Iom Bioworks Pvt. Ltd. and were subsequently processed for DNA extraction. The stabilization conditions and handling procedures were standardized across samples. Microbial DNA was extracted using the QIAamp PowerFecal Pro DNA Kit (Qiagen, NewDelhi, India) following the manufacturer’s guidelines. DNA yield and purity were measured using a NanoDrop One spectrophotometer and a Qubit fluorometer (hermo Fisher Scientific, Waltham, MA, USA), and DNA integrity was confirmed through agarose gel electrophoresis.

### 2.4. Library Preparation and Whole-Metagenome Sequencing

Sequencing libraries were generated using the QIAseq FX DNA Library Kit UDI-C (Qiagen, Hilden, Germany) in accordance with the manufacturer’s protocol. Library concentration and fragment size profiles were evaluated using a Qubit fluorometer and agarose gel electrophoresis. Prepared libraries were then sequenced on the Illumina NovaSeq 6000 platform (Illumina, San Diego, CA, USA) using 150 bp paired-end chemistry, yielding roughly 20 million reads per sample.

### 2.5. Bioinformatic Processing and Taxonomic Profiling

Raw metagenomic reads were processed using the BioBakery workflow. Quality control and removal of sequencing artifacts were performed using KneadData v0.11.0 prior to downstream analysis. Host-derived reads were removed during the quality-control workflow, and the resulting non-host reads were used for microbial taxonomic profiling. Taxonomic composition was determined using MetaPhlAn v4.0.3, which assigns reads to microbial clades by aligning them against a database of taxon-specific marker genes via Bowtie2. This approach produced relative abundance profiles across multiple taxonomic ranks, enabling high-resolution characterization of the microbial community.

### 2.6. Standardization, Composite Domain Scoring, and Multidimensional Physiological Health Index (MPHI) Calculation

Z-score standardization of parameters: All biochemical, physiological, and psychometric variables were standardized before constructing composite indices. Each parameter was converted to a Z-score using Equation (1):

(1) $$Z=\frac{x-\mu }{\sigma }$$

where *x* is the observed value, *μ* is the sample mean, and *σ* is the standard deviation. Standardization placed variables with different units and scales onto a common metric (mean = 0, SD = 1), enabling aggregation across heterogeneous clinical domains.

Composite domain score calculation: Domain-specific indices (metabolic, inflammatory, hepato-renal, psychological well-being, and sleep) were computed using standardized Z-scores for all parameters. They were then derived by summing Z-scores according to physiological direction: variables where higher values reflect better health were included as positive contributors, while variables where higher values reflect poorer health were treated as negative contributors. Domain scores were calculated using Equation (2):

(2) $$\mathrm{Composite}\;\mathrm{Domain}\;\mathrm{Score}\;=\frac{\sum \mathrm{Zpositive}-\sum \mathrm{Znegative}}{n}$$

where *n* is the total number of parameters within the domain. Higher scores indicate better function or lower aging burden; lower scores indicate greater physiological stress. The polarity-adjusted composite scoring method used in this study is widely applied in multidimensional health assessments, including SF-36 [21] and WHOQOL [22], and follows approaches used in composite biomedical indices and frailty models [23,24].

Multidimensional physiological health index (MPHI) construction: A global MPHI was derived to integrate multisystem physiological performance across metabolic, inflammatory, hepato-renal, psychological well-being, and sleep-related domains, providing a multidimensional assessment of physiological health in apparently healthy aging. Each domain was assigned a biologically informed, a priori weight based on published evidence regarding the relevance of metabolic, inflammatory, hepato-renal, psychological, and sleep-related processes to physiological health and apparently healthy aging [23,24,25,26]. The weighting scheme was intended as a structured analytical framework and should not be interpreted as an empirically optimized or clinically validated weighting model. To evaluate the robustness of the proposed weighting scheme, sensitivity analyses were performed using an equal-weight model (20% contribution from each domain) and a perturbed-weight model (30% metabolic, 30% inflammatory, 20% hepato-renal, 10% psychological, and 10% sleep). Agreement between the original and alternative weighting strategies was assessed using Pearson correlation analysis. The equal-weight model was included to evaluate whether the principal findings were dependent on the proposed biologically informed weighting strategy, while the perturbed-weight model assessed the consistency of the MPHI to reasonable variations in domain weighting. The MPHI was calculated using Equation (3).

(3) MPHI = (0.35 × Metabolic) + (0.25 × Inflammatory) + (0.20 × Hepato-Renal) + (0.10 × Psychological) + (0.10 × Sleep)

Metabolic and inflammatory domains were assigned greater weight (35% and 25%, respectively) based on their established relevance to systemic metabolic regulation, inflammatory status, and aging biology. Hepato-renal function received moderate weight (20%) based on its contribution to systemic metabolic and physiological homeostasis [27]. Psychological well-being and sleep quality were weighted at 10% each, based on their established relevance to stress physiology, overall well-being, and systemic physiological regulation. All domain inputs were standardized Z-scores derived from the present study cohort; therefore, the resulting MPHI provides a relative scale for comparison among participants within this cohort. Higher MPHI values indicate a more favorable multidimensional physiological profile, whereas lower values indicate greater physiological dysregulation across the assessed domains. The MPHI is intended as a general multidimensional physiological health index and should not be interpreted as a direct measure or validated surrogate of mitochondrial function. The MPHI should be interpreted as a systems-level physiological proxy and not as a direct measure of mitochondrial function.

### 2.7. Statistical Analysis

All statistical analyses were carried out using JMP Pro (SAS Institute Inc., Cary, NC, USA). Continuous variables are reported as mean ± standard deviation (SD) or median with interquartile range, depending on distribution. Comparisons of continuous clinical, metabolic, inflammatory, and well-being variables across the age–sex groups were performed using appropriate statistical tests based on data distribution. For normally distributed variables, one-way analysis of variance (ANOVA) followed by Tukey’s HSD post hoc test was used, whereas non-normally distributed variables were analyzed using the Kruskal–Wallis test followed by Dunn’s post hoc test. Pairwise post hoc comparisons were used to identify specific group differences when the overall test was significant.

The independent and combined effects of age category (middle-aged vs. elderly) and sex on the physiological domain scores were assessed using two-way factorial ANOVA, including age, sex, and the age × sex interaction as fixed effects. Partial eta-squared (η^2^p) was reported as a measure of effect size for the age, sex, and age × sex interaction effects. Estimated mean differences and their 95% confidence intervals (CIs) were reported for the age and sex effects. Model assumptions were assessed using appropriate diagnostic procedures. Microbiome α-diversity (Shannon and Simpson indices) was calculated to evaluate within-sample richness and evenness. β-diversity was estimated using Bray–Curtis dissimilarity and visualized via Principal Coordinates Analysis (PCoA). Microbiome α-diversity (Shannon and Simpson indices) was calculated to evaluate within-sample richness and evenness. β-diversity was estimated using Bray–Curtis dissimilarity and visualized via Principal Coordinates Analysis (PCoA). Differences in gut microbial community structure between the age–sex groups were tested using Permutational Multivariate Analysis of Variance (PERMANOVA) based on the Bray–Curtis dissimilarity matrix. Multivariate differences in physiological domain scores were assessed separately using multivariate analysis of variance (MANOVA). Associations between gut microbial taxa and biochemical parameters, including metabolic, inflammatory, hepato-renal, psychological, and sleep-related scores, were evaluated using Spearman’s rank correlation analysis, and multivariate regression models were applied where appropriate. To account for multiple hypothesis testing in the microbiome–host correlation analyses, *p*-values were adjusted using the Benjamini–Hochberg false discovery rate (FDR) procedure, and correlations with FDR-adjusted q-values < 0.05 were considered statistically significant. Principal component loadings were examined to determine the contribution of individual physiological domains to the observed multivariate patterns. Unless otherwise specified, all statistical tests were two-tailed, and *p* < 0.05 was considered statistically significant.

## 3. Results

### 3.1. Baseline Host Characteristics in the Apparently Healthy Aging Study Population

Table 1 summarizes the clinical, metabolic, inflammatory, and well-being profiles of middle-aged (40–59 years) and elderly (≥60 years) participants. Across demographic parameters, the mean age was 47.92 ± 5.4 and 48.55 ± 6.8 years for middle-aged males and females, respectively, and 66.48 ± 5.14 and 67.13 ± 8.06 years for elderly males and females. Body mass index (BMI) was similar across all groups (24.9–25.3 kg/m^2^), with group means close to or slightly above the upper end of the normal range. Hematological parameters showed significant differences in hemoglobin levels across the four groups (*p* < 0.001), with higher hemoglobin levels in middle-aged males than middle-aged females (15.1 vs. 12.3 g/dL), whereas elderly males and females showed values of 14.29 and 13.80 g/dL, respectively. Metabolic markers, including fasting blood sugar and HbA1c, did not differ significantly across the groups and remained within or close to normal ranges, but serum uric acid showed a significant group difference (*p* = 0.014), with the highest mean value observed in middle-aged males (6.14 ± 1.60 mg/dL). Lipid profiles indicated a significant difference in HDL-C across the groups (*p* = 0.002), whereas triglycerides and LDL-C did not differ significantly. Inflammatory markers showed no significant difference in CRP (*p* = 0.484), whereas ESR differed significantly across the groups (*p* = 0.012). SGOT and SGPT were within normal limits across all groups, with no significant group differences. Blood urea and serum creatinine levels were within normal ranges, with no clinically significant differences between age or sex groups. Among the well-being and sleep measures, global PSQI scores differed significantly across the groups (*p* = 0.022), with the lowest mean score observed in elderly males (2.75 ± 1.54) and the highest in elderly females (8.50 ± 4.38). The WHOQOL physical domain also showed a significant group difference (*p* = 0.014), whereas psychological and social functioning did not differ significantly across the groups. SF36 energy/fatigue scores were comparable across all groups (*p* = 0.103), indicating generally preserved vitality.

### 3.2. Z-Score Distribution Across Clinical, Metabolic, Inflammatory, Hepato-Renal, Psychological, and Sleep Parameters

**Figure 2:**
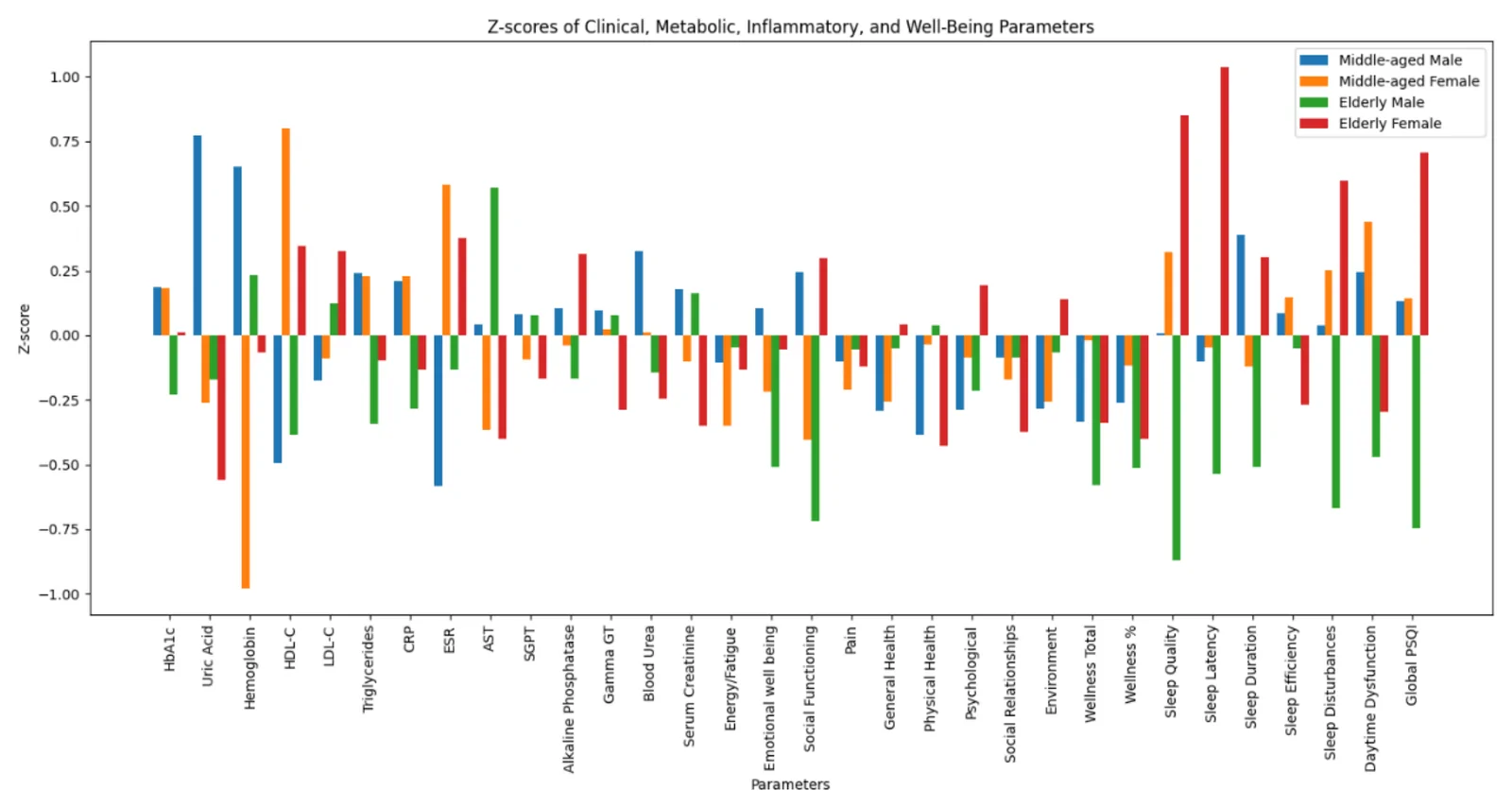
The distribution of standardized Z-scores for clinical, metabolic, inflammatory, hepato-renal, psychological, and sleep parameters across middle-aged males, middle-aged females, elderly males, and elderly females.

Z-scores for clinical, metabolic, inflammatory, and well-being parameters were calculated and the grouped bar plot (Figure 2) shows the distribution of Z-scores across the four participant groups. Middle-aged males had slightly elevated HbA1c Z-scores (0.19) and uric acid Z-scores (0.77), indicating a tendency toward higher metabolic risk, whereas middle-aged females had lower uric acid Z-scores (−0.26) but lower hemoglobin Z-scores (−0.98), reflecting sex-related differences in hematology and metabolic health. HDL-C Z-scores were lower in middle-aged males (−0.50), reflecting reduced cardioprotective lipid levels, while LDL-C Z-scores were elevated in elderly participants (male: 0.12, female: 0.33), consistent with age-related increases in atherogenic lipids. Triglyceride Z-scores were modestly higher in middle-aged males (0.24) than elderly participants. Hemoglobin Z-scores were higher in middle-aged males (0.65) and lower in middle-aged females (−0.98), reflecting normal sex-related physiological differences. CRP and ESR Z-scores were slightly elevated in middle-aged males and females (CRP: ~0.21–0.23; ESR: −0.58 to 0.58), suggesting mildly increased inflammatory burden, whereas elderly participants showed slightly lower inflammatory Z-scores. The Z-scores calculated for SGOT, SGPT, alkaline phosphatase, gamma-GT, blood urea, and serum creatinine were within ±0.5, indicating no major hepatic or renal deviations. Energy/fatigue and psychological Z-scores were largely comparable across groups. Elderly females showed reduced social functioning Z-scores (−0.37), suggesting age-related declines in social engagement. Global PSQI Z-scores indicated poorer sleep in middle-aged males (0.39) and relatively better sleep indices in elderly females (0.70). Figure 2 highlights parameter-specific risk patterns. Middle-aged males tend to show higher metabolic and inflammatory Z-scores, indicating elevated risk, whereas elderly participants, particularly females, show lower social and psychological scores, consistent with age-related decline in certain well-being domains.

### 3.3. Multidimensional Health Indexing Reveals Differential Metabolic and Functional Aging Patterns in Middle-Aged and Elderly Adults

**Figure 3:**
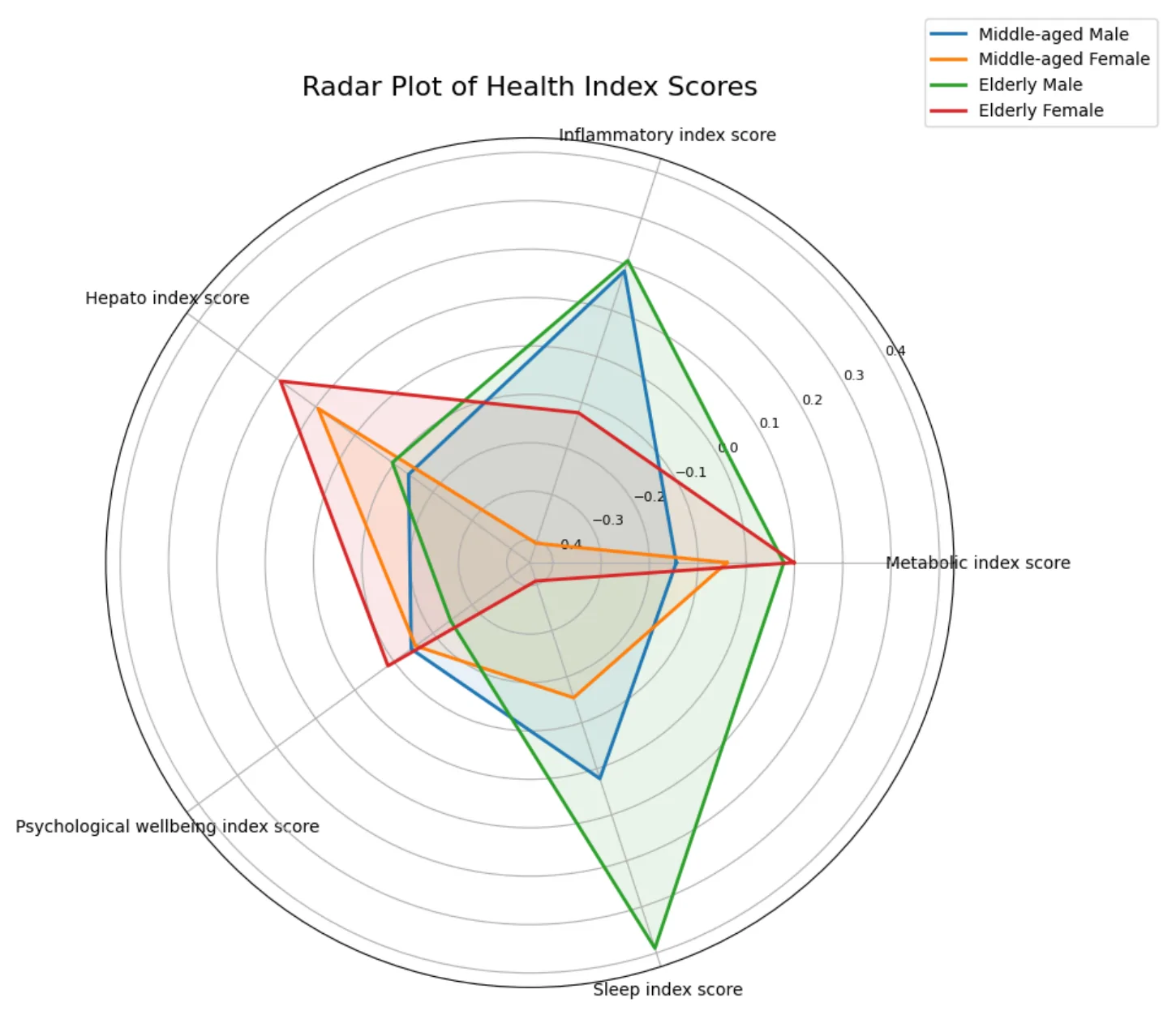
Radar plot comparing composite physiological domain scores (metabolic, inflammatory, hepato-renal, psychological, and sleep) among the four age–sex groups.

**Figure 4:**
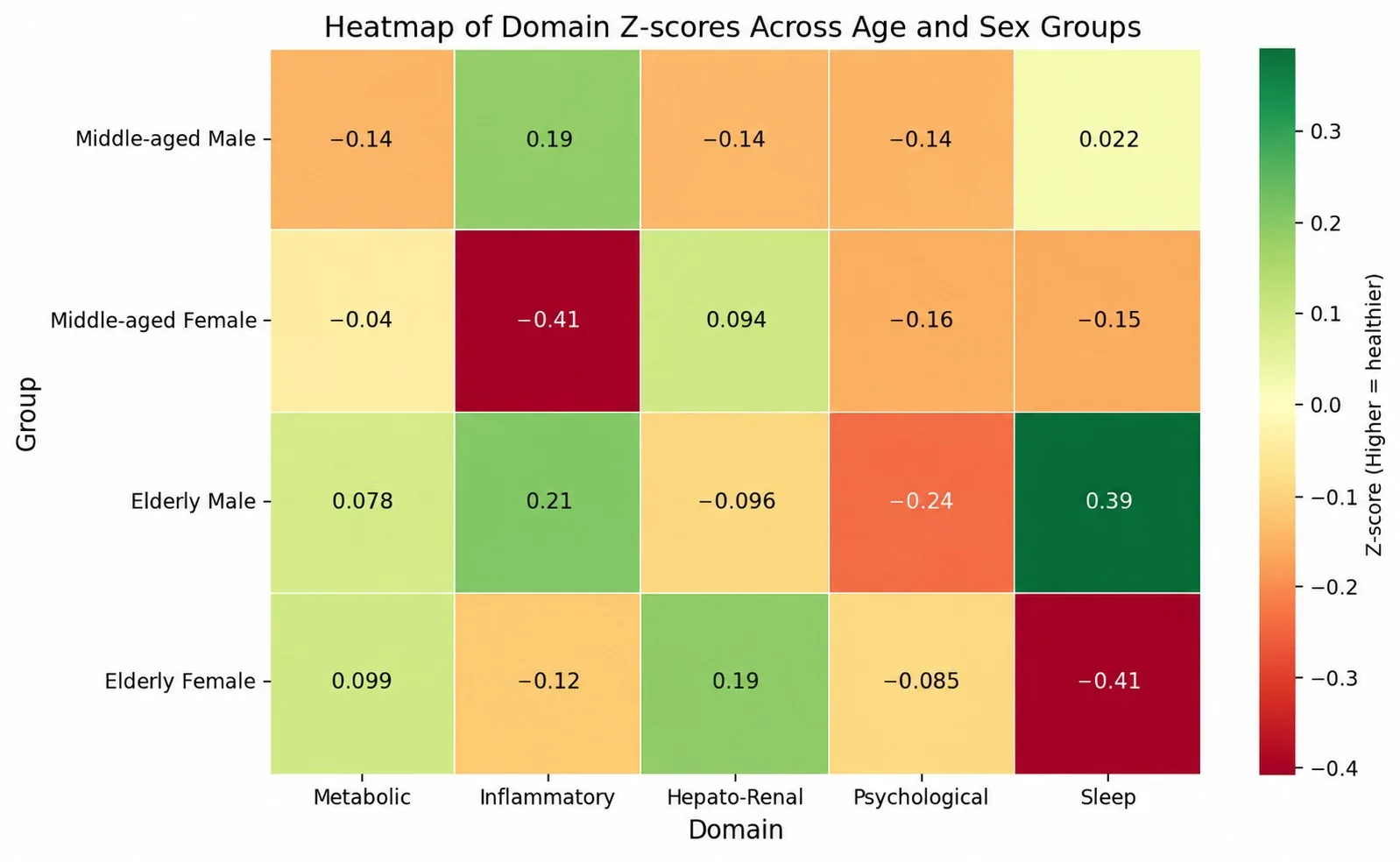
Heatmap of composite physiological domain Z-scores across age and sex groups. Higher Z-scores (green) indicate comparatively more favorable physiological status, whereas lower Z-scores (red) indicate comparatively less favorable physiological status. The color scale is centered at zero (yellow).

We computed composite domain scores for metabolic, inflammatory, hepato-renal, psychological well-being, and sleep indices based on the multi-domain Z-score analysis. These scores were visualized using a radar plot (Figure 3), providing an intuitive overview of group-specific health profiles. In addition, a heatmap (Figure 4) shows the direction and magnitude of each domain score between groups, making it easier to compare relative strengths and deficits. Higher scores in this graphic show improved health or a lesser risk of aging, whereas lower (negative) scores show functional deterioration or weaknesses in the corresponding domain. Elderly females (0.10) and elderly males (0.08) showed slightly positive metabolic index scores, suggesting relatively preserved metabolic function, whereas middle-aged males (−0.14) and females (−0.04) displayed slightly lower scores. Middle-aged males (0.19) and elderly males (0.21) had higher inflammatory index scores, indicating better inflammatory status, while middle-aged females (−0.41) and elderly females (−0.12) had negative scores, highlighting increased inflammatory risk. Elderly females (0.19) and middle-aged females (0.09) showed positive hepato-renal index scores, suggesting preserved liver and kidney function, whereas middle-aged males (−0.14) and elderly males (−0.10) had slightly negative scores. All groups showed mild negative psychological well-being index scores, with elderly males (−0.24) and middle-aged females (−0.16) showing the largest declines, reflecting lower mental health and social functioning. Elderly males had the highest score (0.39), indicating better sleep quality, whereas elderly females (−0.41) and middle-aged females (−0.15) had negative sleep index scores, suggesting poorer sleep quality in these groups.

**Figure 5:**
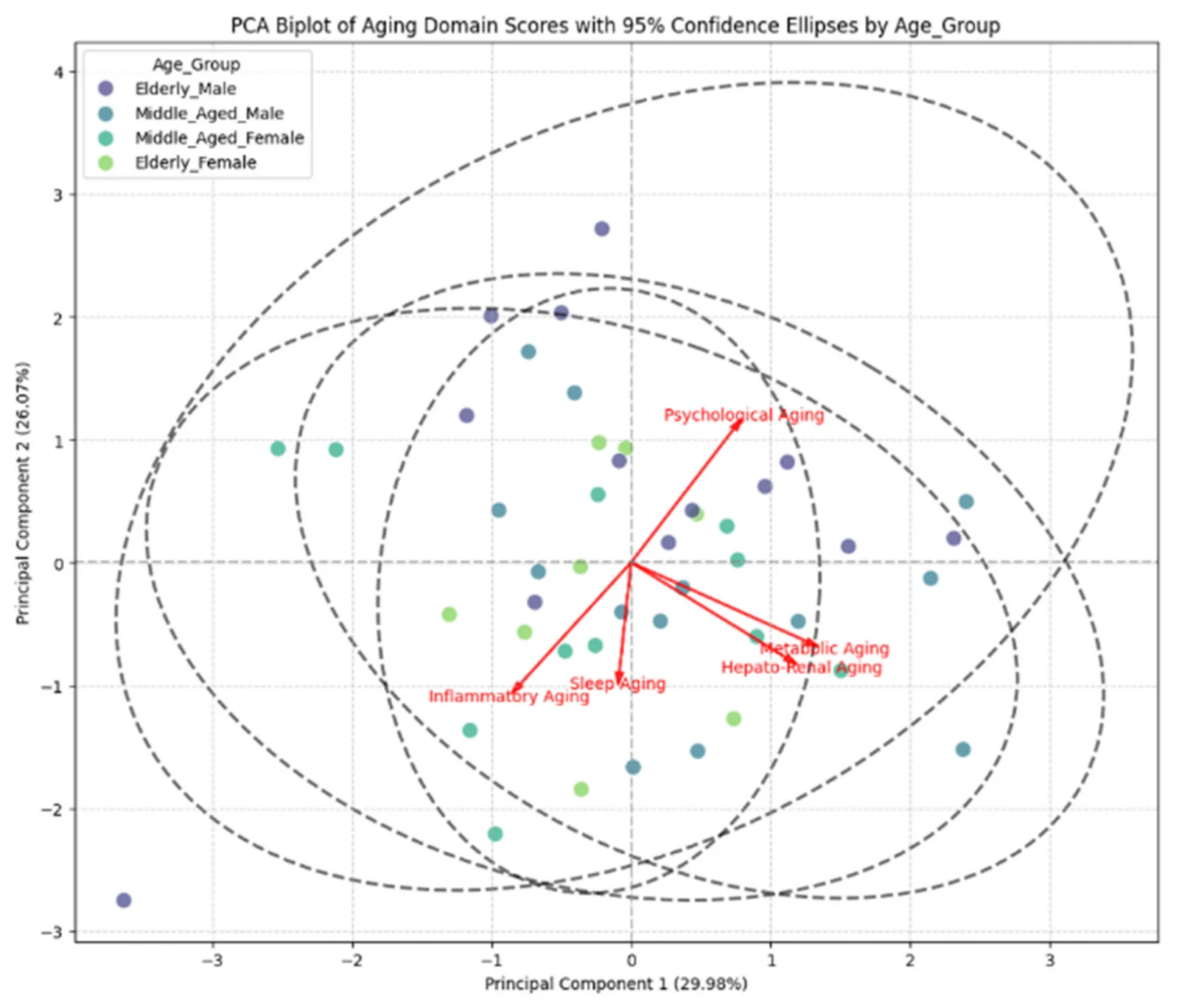
Principal component analysis (PCA) of the five composite physiological domain scores (metabolic, inflammatory, hepato-renal, psychological, and sleep). The PCA plot includes 95% confidence ellipses for each age–sex group. PC1 and PC2 explained 29.98% and 26.07% of the total variance, respectively.

Principal component analysis (PCA) was performed to examine multivariate relationships among the five composite domain scores (metabolic, inflammatory, hepato-renal, psychological well-being, and sleep) across the four age-and sex-defined groups. The first two principal components explained 56.05% of the total variance, with PC1 accounting for 29.98% and PC2 accounting for 26.07% (Figure 5). The PCA biplot showed substantial overlap among the 95% confidence ellipses of the four groups, indicating limited separation of the multidimensional physiological profiles. The PCA loading matrix (Table 2) indicated that PC1 was primarily influenced by the metabolic (loading = 0.629) and hepato-renal (loading = 0.555) domains, whereas PC2 was characterized by a positive loading for the psychological domain (loading = 0.546) and negative loadings for the inflammatory (loading = −0.497) and sleep (loading = −0.449) domains. Multivariate analysis using MANOVA showed no statistically significant differences among the four groups (Wilks’ λ = 0.570, F = 1.544, *p* = 0.103).

### 3.4. Two-Way ANOVA of Physiological Domain Scores by Age and Sex

Two-way factorial ANOVA was performed to assess the independent effects of age category and sex, as well as their interaction, on the five physiological domain scores and the MPHI (Table 3). No significant age effects were observed for the metabolic, inflammatory, hepato-renal, psychological, sleep, or MPHI scores (all *p* > 0.05). A significant main effect of sex was observed for the inflammatory domain (*F*(1,41) = 4.97, *p* = 0.031, η^2^p = 0.108) and sleep domain (*F*(1,41) = 6.60, *p* = 0.014, η^2^p = 0.139). A significant age × sex interaction was also observed for the sleep domain (*F*(1,41) = 6.44, *p* = 0.015, η^2^p = 0.136). No significant age × sex interactions were observed for the metabolic, inflammatory, hepato-renal, psychological, or MPHI scores (all *p* > 0.05). The MPHI itself showed no significant main effect of age (*p* = 0.092), sex (*p* = 0.241), or age × sex interaction (*p* = 0.334). These findings indicate that although some domain-specific differences were associated with sex and the combined effects of age and sex, the overall MPHI did not differ significantly across age or sex categories.

### 3.5. Composite Multidimensional Physiological Health Index (MPHI) Across Age and Sex

**Figure 6:**
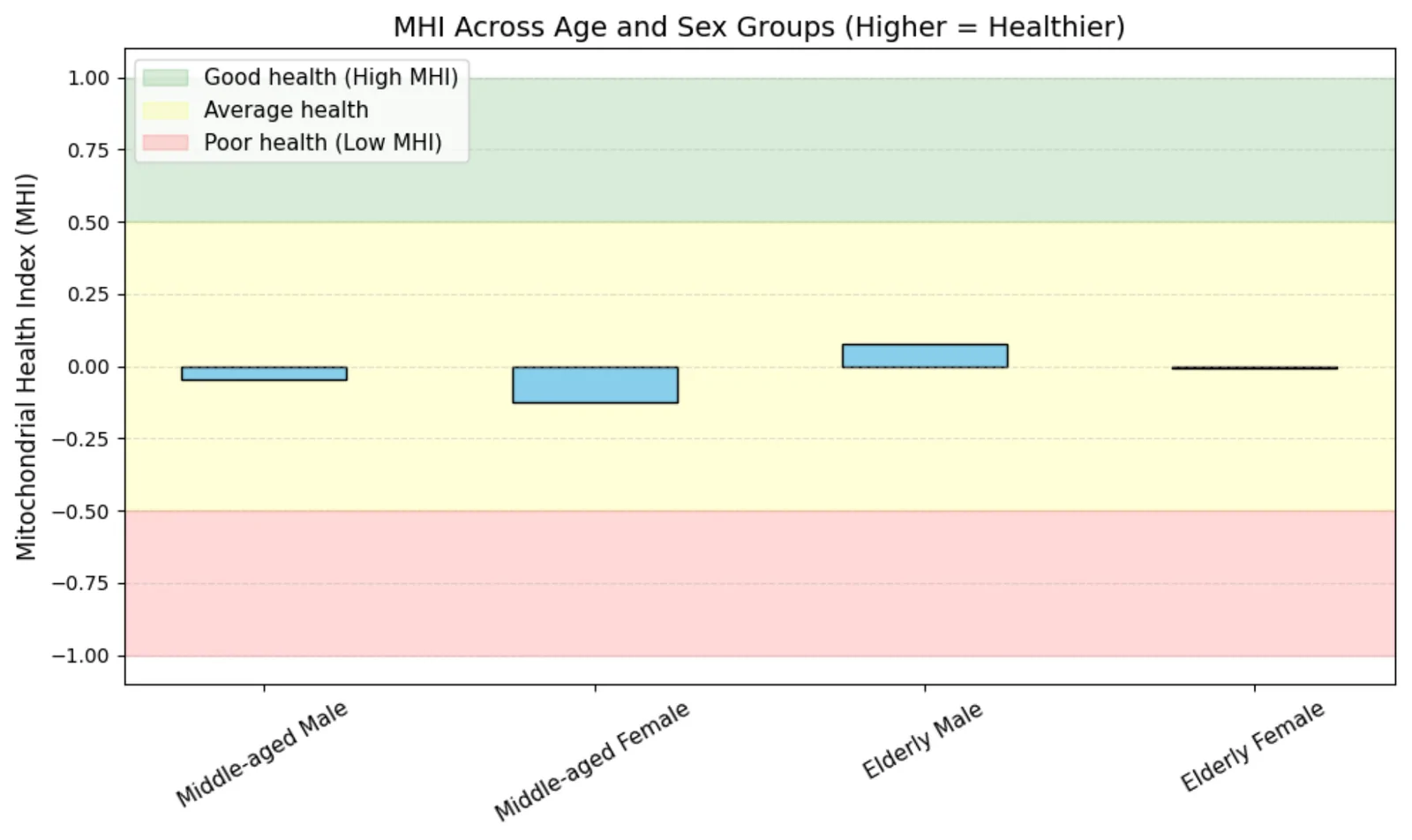
Comparison of the composite multidimensional physiological health index (MPHI) across middle-aged and elderly males and females.

To provide an integrated assessment of multidimensional physiological status, we calculated the composite MPHI from the composite domain scores. The MPHI integrates five systemic domains, i.e., metabolic, inflammatory, hepato-renal, psychological well-being, and sleep, that are individually associated with mitochondrial physiology and apparently healthy aging, providing a systems-level physiological proxy rather than a direct measure of mitochondrial function [28,29]. The computed MPHI values showed numerical variation across the four age–sex groups (Figure 6). The highest MPHI was seen among elderly men (0.0747), followed by elderly women (−0.0072). Middle-aged females had the lowest MPHI (−0.1275). Middle-aged males displayed an intermediate MPHI score (−0.0440). These findings reflect differences in the integrated physiological profiles captured across the five domains.

**Figure 7:**
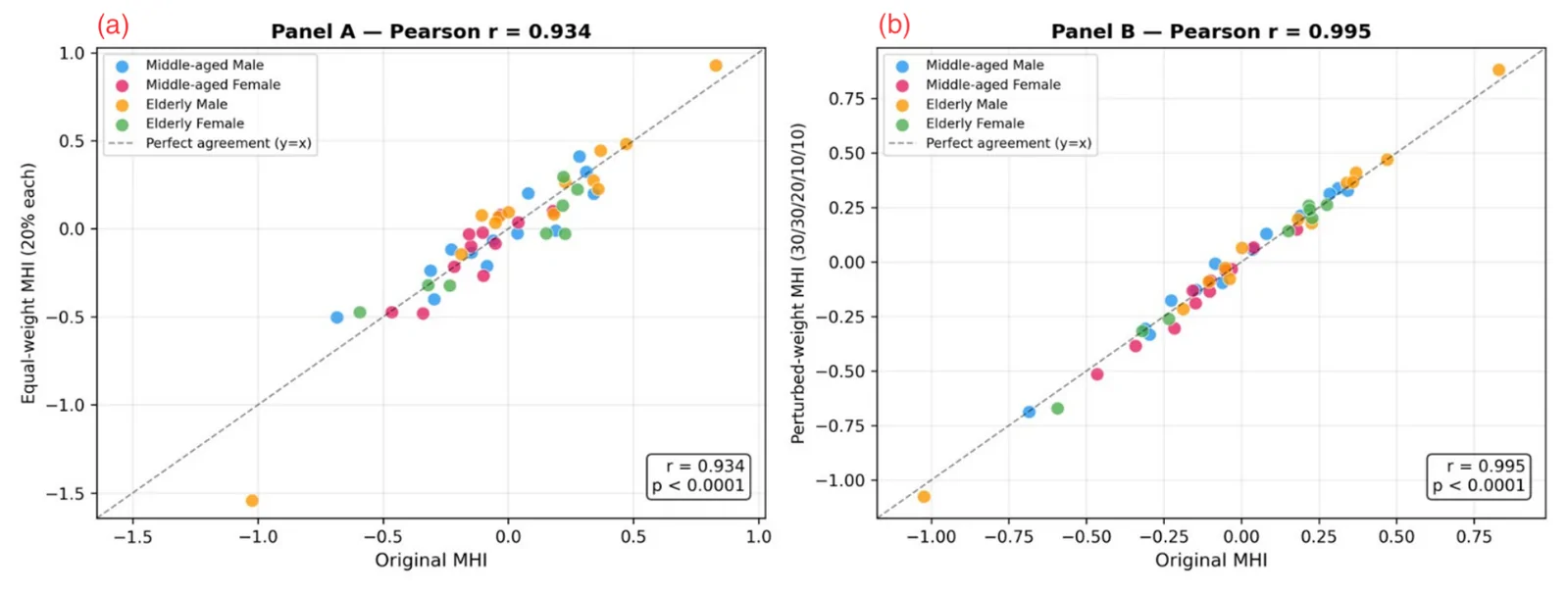
(**a**) Comparison of MPHI values generated using the original and equal-weight models. (**b**) Comparison of MPHI values generated using the original and perturbed-weight models.

To evaluate the stability of the proposed weighting scheme, sensitivity analyses were performed using equal-weight and perturbed-weight models. MPHI values generated using the alternative weighting strategies showed strong agreement with the original MPHI (original vs. equal-weight model: Pearson’s r = 0.934, *p* < 0.0001; original vs. perturbed-weight model: Pearson’s r = 0.995, *p* < 0.0001; Figure 7a,b). Across all weighting strategies, elderly males consistently exhibited the highest MPHI values and middle-aged females the lowest (Table 4), indicating that the principal findings were robust to reasonable variations in domain weighting and were not dependent on the original biologically informed weighting scheme.

### 3.6. Microbial Diversity and Community Structure in Middle-Aged and Elderly Adults

**Figure 8:**
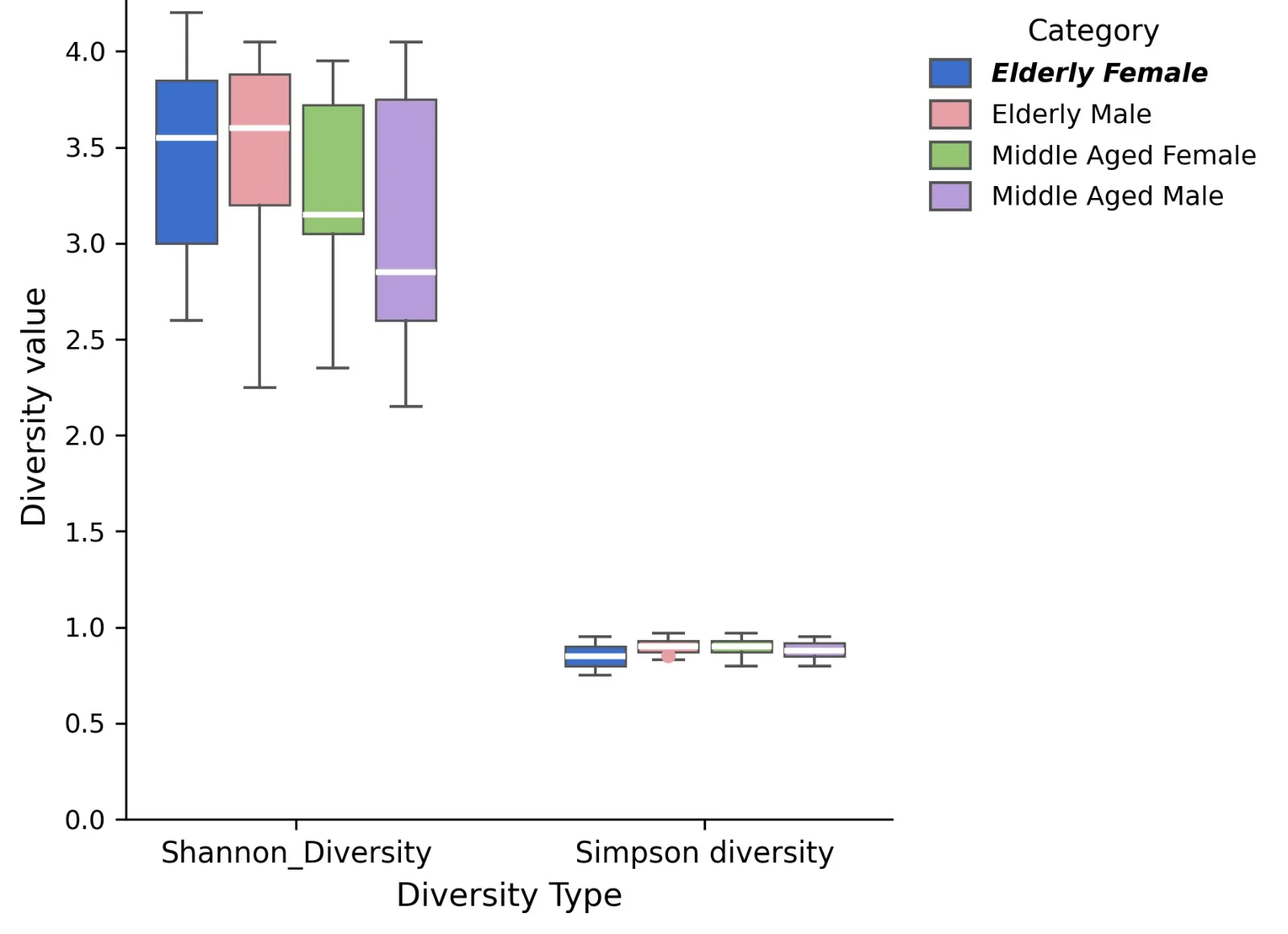
Alpha diversity of the gut microbiome across age and sex groups, showing microbial richness and diversity indices.

**Figure 9:**
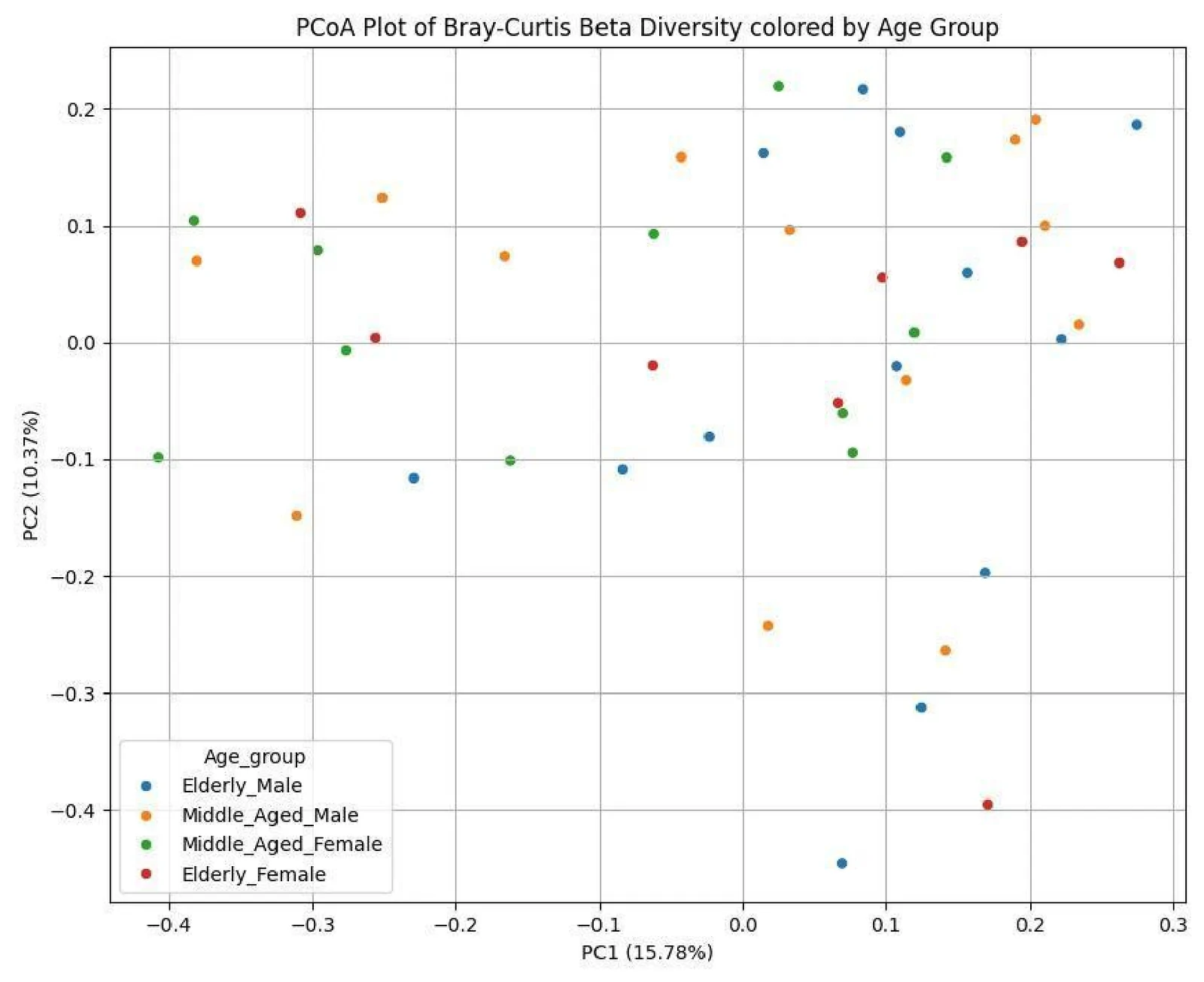
Principal Coordinates Analysis (PCoA) based on Bray–Curtis dissimilarity showing the overall similarity of gut microbial community composition among the four age–sex groups.

Taxonomic richness varied across the age–sex groups, with 13–15 phyla, 47–48 orders, 97–106 families, 373–447 genera, and 898–987 species identified across the groups (Table 5). This pattern of stability was further supported by Alpha diversity measurements (Figure 8), which revealed overlapping distributions across groups in both the Shannon and Simpson indices, with no statistically significant difference between middle-aged and elderly males and females. Beta diversity analysis using Bray–Curtis distances demonstrated a high degree of compositional overlap, with PCoA plots (Figure 9) showing no group-wise clustering. PERMANOVA based on the Bray–Curtis dissimilarity matrix also showed no statistically significant differences in gut microbial community structure among the four age–sex groups (Pseudo-F = 0.933, R^2^ = 0.0639, *p* = 0.651), indicating that the defined age–sex groups explained only 6.39% of the variation in gut microbial community composition. Samples from all four demographic categories were interspersed, indicating that inter-individual variability exceeded group-level differences.

**Figure 10:**
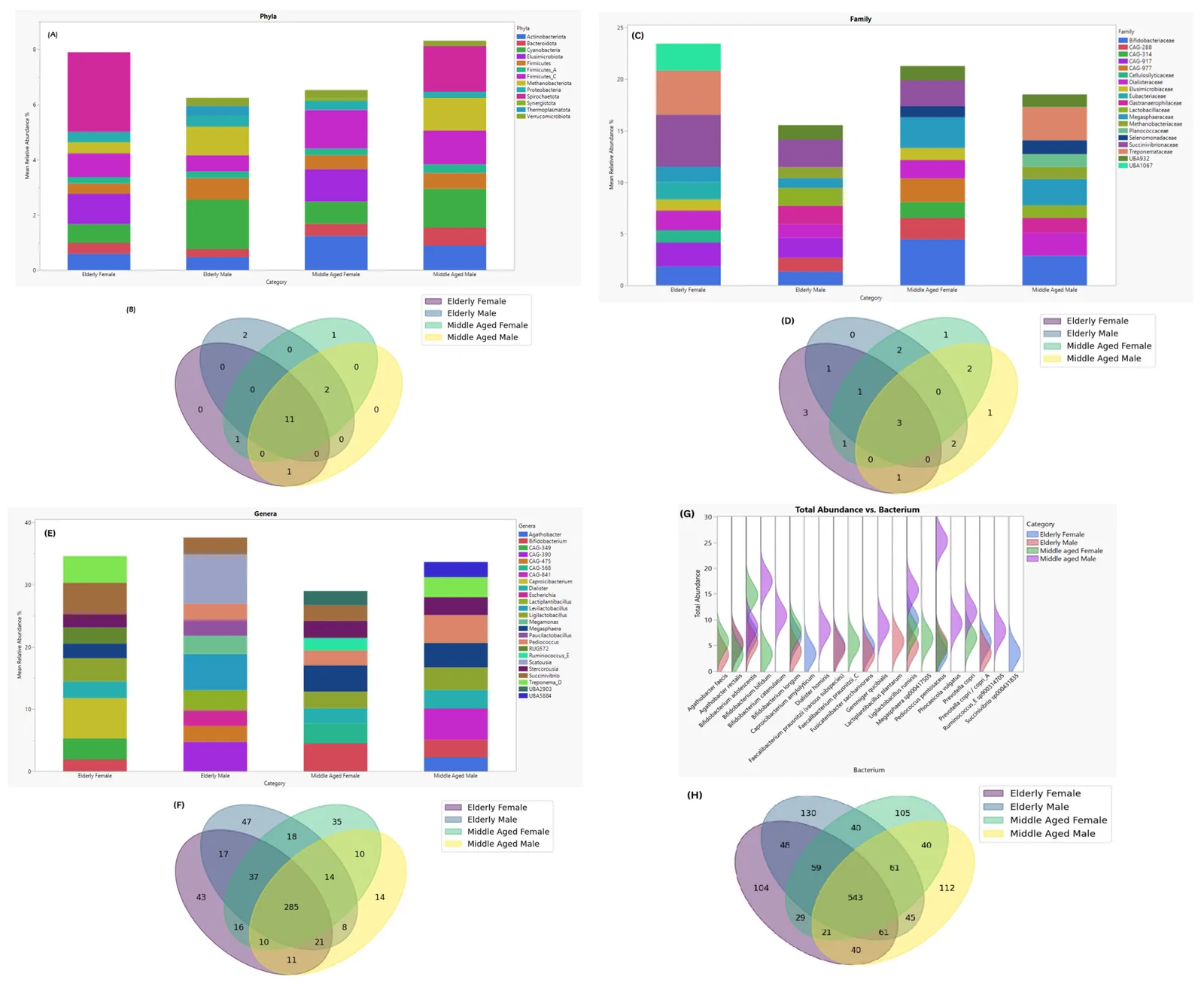
Gut microbiota composition and shared taxa in age–sex groups at different taxonomic levels. (**A**) Relative abundance of gut microbiota at the phylum level across age–sex groups. (**B**) Venn diagram showing shared and unique microbial taxa across the four demographic groups. (**C**) Relative abundance of gut microbiota at the family level across age–sex groups. (**D**) Venn diagram showing shared and unique microbial taxa across the four demographic groups. (**E**) Relative abundance of gut microbiota at the genus level across age–sex groups. (**F**) Venn diagram showing shared and unique microbial taxa across the four demographic groups. (**G**) Relative abundance of gut microbiota at the species level across age–sex groups. (**H**) Venn diagram showing shared and unique microbial taxa across the four demographic groups.

The gut microbiota compositional profiles for the middle-aged and elderly groups are as shown in Figure 10. At the phylum level, the top ten phyla were visualized (Figure 10A,B). The dominant phyla detected in all groups were Firmicutes, Bacteroidota, *Actinobacteriota*, and Proteobacteria. Minor phyla including Verrucomicrobiota, *Synergistota*, *Methanobacteriota*, *Cyanobacteria*, *Spirochaetota*, *Elusimicrobiota*, and *Thermoplasmatota* were present at lower but comparable relative abundances. At the family level, *Lactobacillaceae*, *Bifidobacteriaceae*, *Treponemataceae*, *Methanobacteriaceae*, *Dialisteraceae*, *Succinivibrionaceae*, *Planococcaceae*, and *Eubacteriaceae* were the main families in all four demographic groups (Figure 10C,D). Other families that contributed lesser but constant proportions across categories were CAG (288, 314, 917 and 977), *Cellulolyticaceae*, *Selenomonadaceae*, *Gastranerophilaceae* and UBA (932 and 1067). *Agathobacter*, *Bifidobacterium*, *Ligilactobacillus*, *Megamonas*, *Dialister*, *Treponema_D*, *Caproiciproducens*, *Succinivibrio*, *Ruminococcus*, and *Methanobrevibacter* were among the prominent genera found in all groups (Figure 10E,F). Numerous additional genera belonging to *Firmicutes*, *Bacteroidota*, and *Actinobacteriota* were detected at moderate-to-low abundances, contributing to a total of 200 genera consistently observed across groups. The species-level abundance distributions for the middle-aged and elderly groups are presented as a violin plot (Figure 10G,H). Across the four demographic groups, the bacterial species consistently detected included *Agathobacter rectalis*, *Ligilactobacillus ruminis* and *Pediococcus pentosaceus*.

### 3.7. Microbial–Host Physiological Correlation Profiling

**Figure 11:**
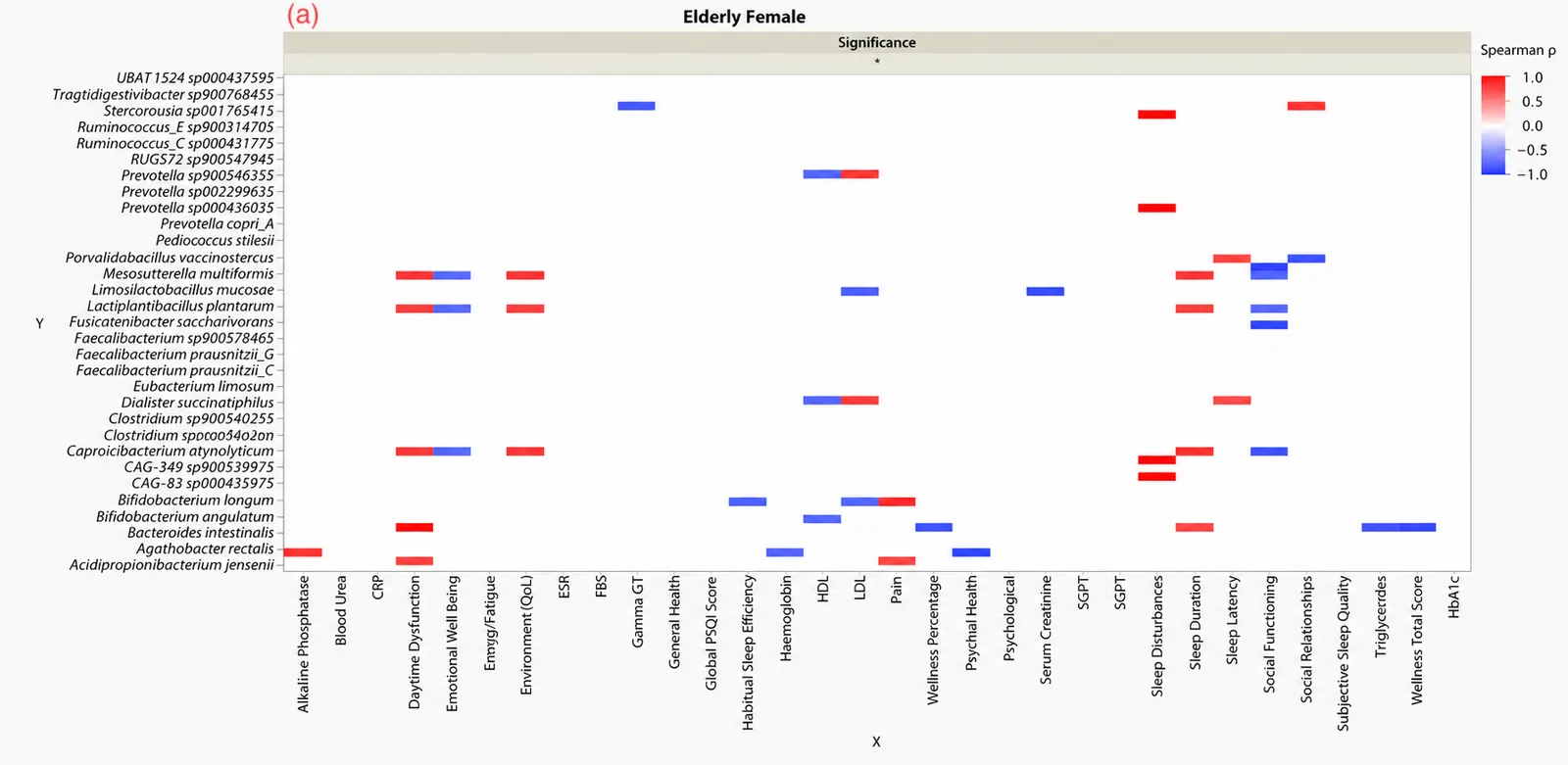

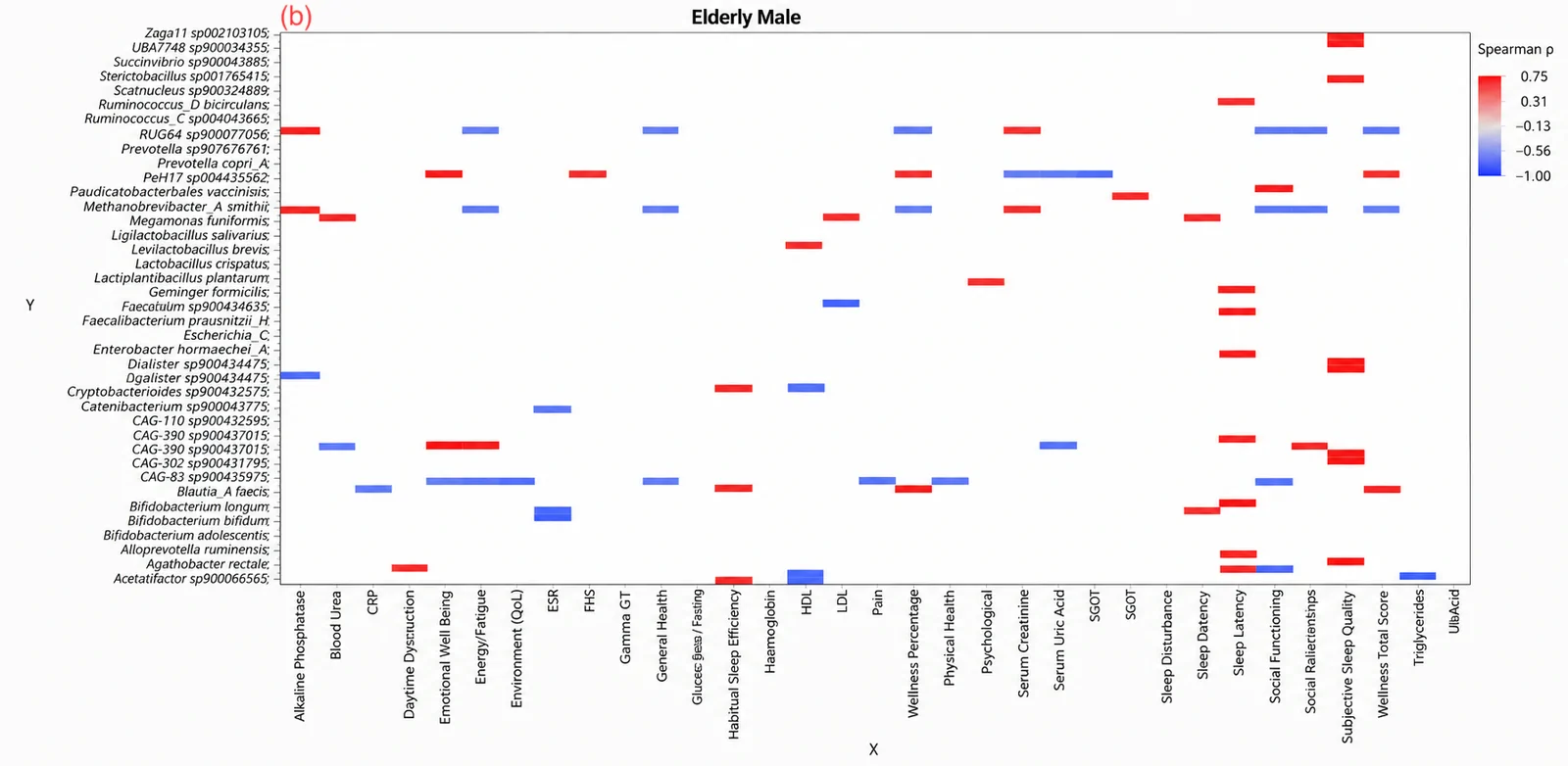

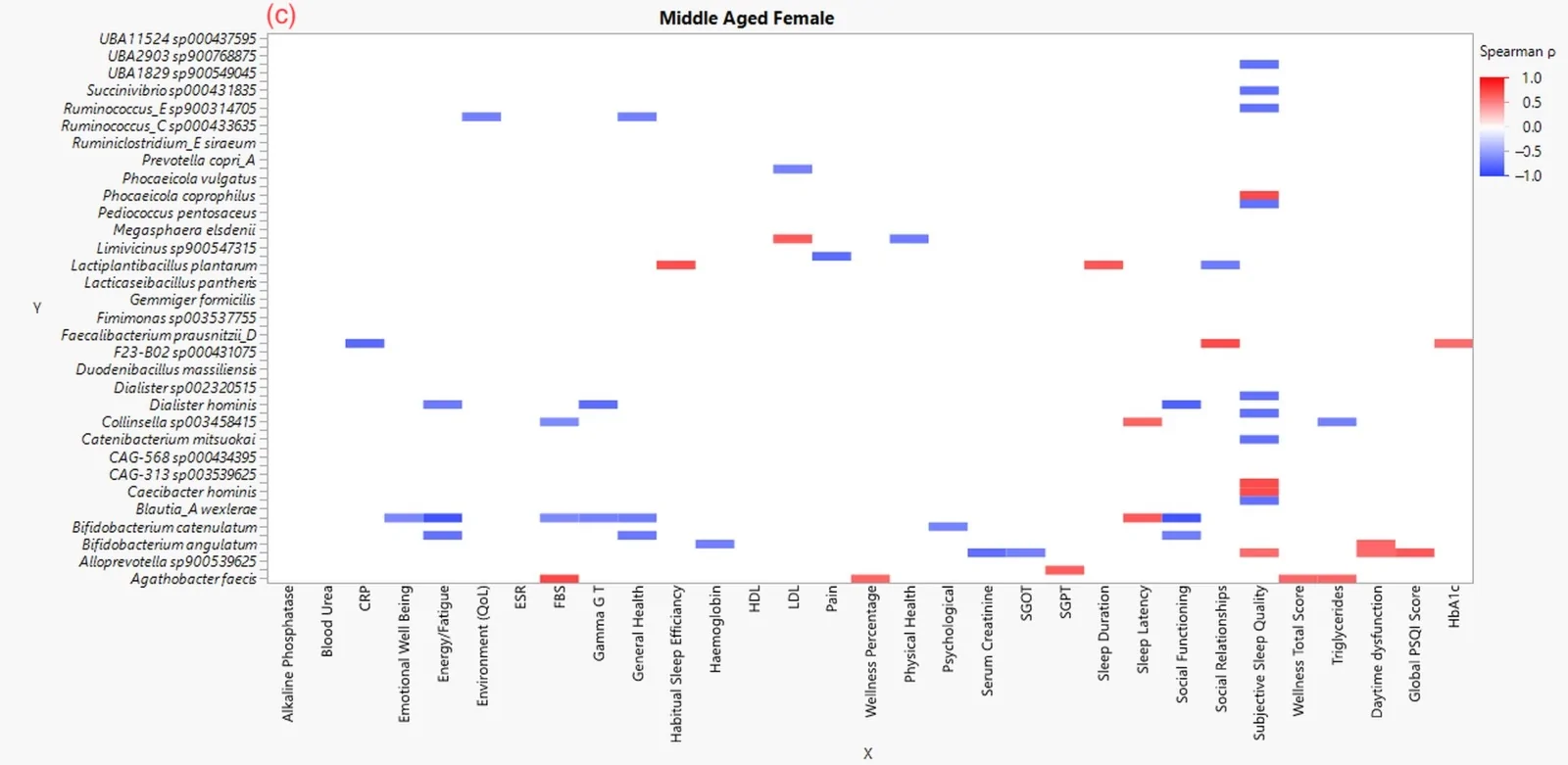

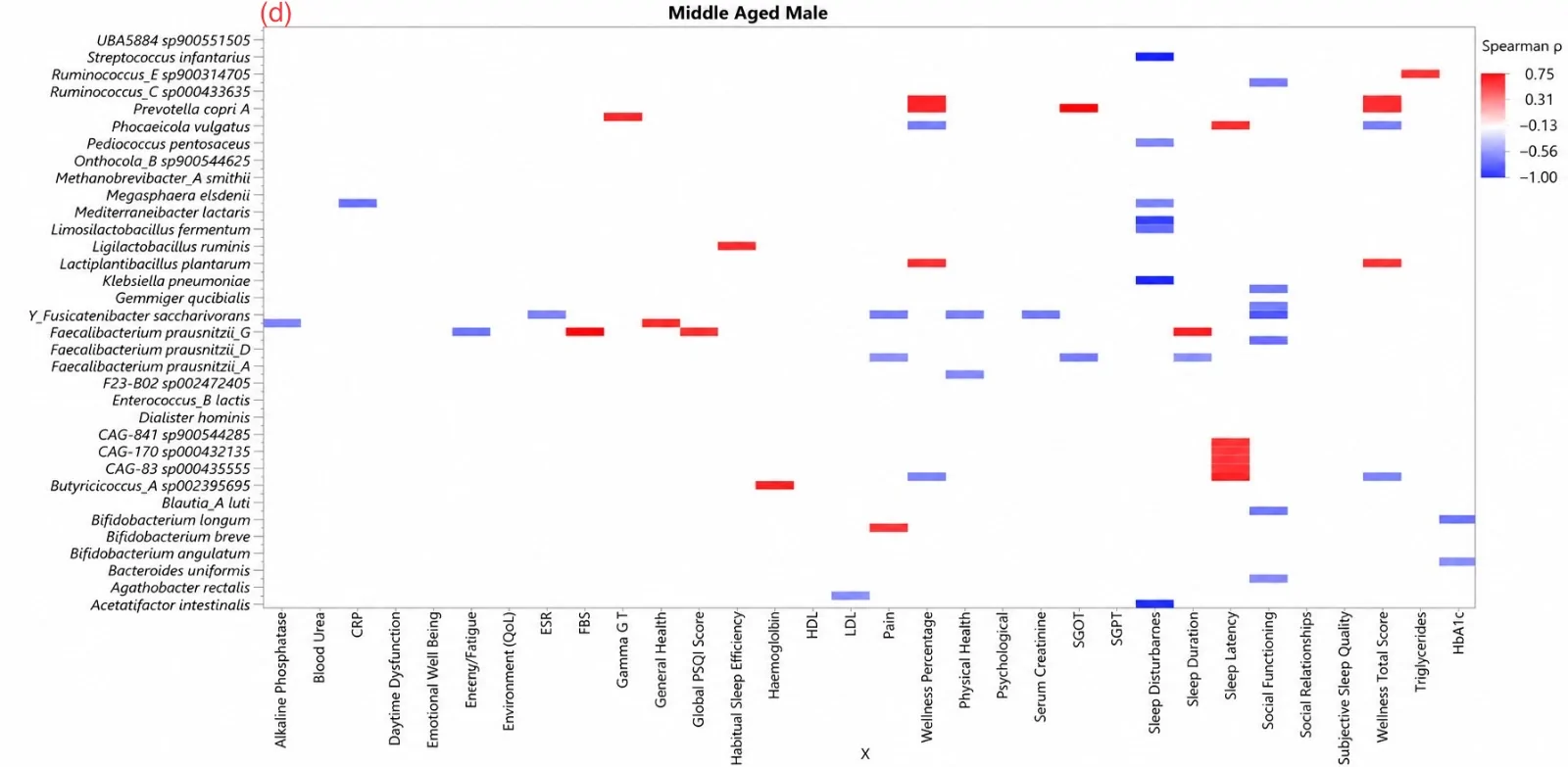
Exploratory Spearman correlation heatmaps showing nominal associations between gut microbial species and host physiological parameters in (**a**) elderly females, (**b**) elderly males, (**c**) middle-aged females, and (**d**) middle-aged males. Positive correlations are shown in red and negative correlations in blue, with color intensity representing the strength of the correlation. None of the associations remained statistically significant after Benjamini–Hochberg false discovery rate correction; therefore, these results should be interpreted as exploratory and hypothesis-generating.

Spearman’s rank correlation analysis was performed to explore relationships between gut microbial species and host physiological parameters across the four study groups. Correlation heatmaps illustrating nominally significant (uncorrected *p* < 0.05) associations are presented in Figure 11a–d, where red indicates positive correlations and blue indicates negative correlations. Following Benjamini–Hochberg false discovery rate (FDR) correction for multiple comparisons, none of the observed correlations remained statistically significant (all q > 0.05). Therefore, the associations described below should be interpreted as exploratory trends rather than statistically confirmed relationships.

The elderly female group demonstrated several nominal positive correlations between specific microbial species and metabolic parameters (Figure 11a). *Bifidobacterium longum* and *Eubacterium limnosum* exhibited clear positive correlations with HDL levels, indicating a nominal positive association with HDL concentrations. In contrast, *Bifidobacterium angulatum* showed a negative correlation with triglycerides. *Bifidobacterium longum* and *Mesosutterella multiformis* were negatively correlated with LDL, whereas *Eubacterium limnosum* demonstrated a negative correlation with LDL. None of the bacterial taxa displayed a significant correlation (positive or negative) with CRP and ESR. For psychological wellness-related measures, *Bacteroides* sp. was negatively correlated with overall wellness percentage, while social functioning was negatively influenced by *Caproicibacterium amylolyticum*, *Lactiplantibacillus plantarum*, *Limosilactobacillus* mucosae, *Pediococcus stilesii*, and *Paucilactobacillus vaccinostercus*. Sleep-related metrics displayed some of the strongest correlations in this group. Several taxa including *Prevotella copri*, *Pediococcus stilesii*, and *Limosilactobacillus* mucosae exhibited nominal positive correlations with sleep latency and sleep disturbances, indicating poorer sleep with higher abundance of these species. Serum creatinine showed a nominal negative correlation with *Mesosutterella multiformis*, whereas alkaline phosphatase demonstrated a positive association with *Agathobacter rectalis*.

In elderly males, several gut microbial taxa showed several nominal correlations with metabolic, heptao-renal, sleep, and psychosocial variables (Figure 11b). *Megamonas* displayed a positive correlation with blood urea, whereas multiple CAG-group taxa were negatively correlated with this marker. Inflammatory markers also exhibited nominal microbial correlations, with *Blautia* positively correlated with CRP. Lipid parameters demonstrated distinct microbial patterns: HDL showed positive associations with *Agathobacter rectalis*, *Acetatifactor* sp., and *Cryptobacteriods* sp., while LDL was positively associated with *Gemmiger formicilis* and negatively with *Methanobrevibacter*. Triglycerides exhibited a negative correlation with *Agathobacter rectalis*. Serum uric acid was negatively correlated with *Prevotella copri* and several CAG taxa. Liver function measures showed targeted relationships, as SGPT was positively correlated with *Paucilactobacillus vaccinostercus*. Pain scores were negatively correlated with CAG-group taxa, while perceived well-being showed positive correlations with *Blautia* and *Prevotella copri* but negative correlations with *Methanobrevibacter* and *Ruminococcus_C*. Sleep-related variables presented multiple associations: sleep duration correlated positively with *Bifidobacterium longum* and *Methanobrevibacter*, and sleep latency showed positive correlations with several species, including *B. longum*, *Escherichia coli*, and various CAG taxa. Social functioning was negatively associated with *Agathobacter rectalis* and CAG taxa. Overall, the elderly male group exhibited numerous nominal microbiota–host correlations across metabolic, inflammatory, sleep, and psychosocial domains; however, none remained statistically significant following FDR correction.

In middle-aged females, CRP showed a nominal negative correlation with *Faecalibacterium prausnitzii_D*, while no microbial species exhibited nominal associations with ESR (Figure 11c). FBS exhibited positive correlations with *Agathobacter faecis* and negative correlations with *Blautia*_A and *Collinsella* sp. Gamma-GT was negatively correlated with both *Blautia*_A and *Dialister* sp., whereas SGPT showed a positive association with *Alloprevotella* sp. Sleep duration demonstrated a positive correlation with *Lactiplantibacillus plantarum*, suggesting a potential link between this species and sleep maintenance. HbA1c was positively associated with *Faecalibacterium prausnitzii_D*. Social functioning showed negative correlations with *Bifidobacterium catenulatum*, *Blautia*_A, and *Dialister* hominis. Social relationship scores were positively associated with *Faecalibacterium prausnitzii_D*, but negatively associated with *Lactiplantibacillus plantarum*. Daytime dysfunction displayed positive correlations with *Alloprevotella* and *Bifidobacterium angulatum*.

In middle-aged males, alkaline phosphatase demonstrated a nominal negative correlation with *Faecalibacterium saccharivorans*, while CRP was nominally negatively correlated with *Megasphaera elsdenii*. Energy and fatigue scores showed a nominal negative association with *Faecalibacterium prausnitzii_G*, and ESR was negatively correlated with *Gemmiger formicilis*. Fasting blood FBS displayed a nominal positive correlation with *Faecalibacterium prausnitzii_G*, whereas Gamma-GT showed a nominal positive association with *Prevotella copri*. Pain scores showed a nominal positive association with *Bifidobacterium longum* but were nominally negatively correlated with *Faecalibacterium prausnitzii_D* and *Gemmiger formicilis*. SGPT levels were nominally positively associated with *Prevotella copri_A* and nominally negatively associated with *Faecalibacterium prausnitzii_D*. Sleep disturbances involved numerous microbial associations, including notable positive correlations with *Acetatifactor intestinalis* and *Streptococcus infantarius*, while sleep latency was nominally positively correlated with CAG-group taxa and *Phocaeicola vulgatus*. Triglyceride levels were positively associated with *Ruminococcus_Esp*, and social functioning scores showed nominal negative correlations with multiple taxa, including *Blautia*, *Gemmiger formicilis*, and *Ruminococcus_Esp* (Figure 11d). Although numerous nominal correlations were observed between gut microbial species and host physiological parameters across the four demographic groups, none remained statistically significant after Benjamini–Hochberg FDR correction (all q > 0.05). These findings therefore represent exploratory observations that may inform future hypothesis-driven studies in larger cohorts.

## 4. Discussion

### 4.1. Microbial–Host Physiological Associations in Apparently Healthy Aging

The study assessed clinical, metabolic, inflammatory, and well-being parameters across middle-aged and elderly participants and later integrated these data into a composite MPHI. The majority of metabolic and hematological markers were within normal reference limits, indicating largely intact physiological health, while demographic variables, such as BMI, remained consistent across age and sex groups. However, subtle age- and sex-related differences were observed, including higher serum uric acid and triglyceride levels in middle-aged males and modest reductions in HDL-C in this group. These findings are consistent with previous studies showing that, even in apparently healthy populations, metabolic risk profiles might vary by age and sex [30,31,32].

Inflammatory markers and hepato-renal function tests remained within normal limits, suggesting the absence of overt systemic inflammation or organ dysfunction. However, elderly females showed a reduction in social functioning scores compared with younger participants, reflecting potential age-related psychosocial decline [33]. SF36 energy/fatigue scores were largely preserved across all groups, consistent with stable subjective vitality. Sleep quality appeared slightly better in the elderly compared with middle-aged participants, although this observation may be influenced by behavioral adaptations, survivor bias, or differences in self-reporting [34].

### 4.2. Multidimensional Physiological Health and Aging-Related Patterns

Mitochondrial dysfunction is widely recognized as a central hallmark of aging, influencing multiple physiological systems and contributing to the onset of chronic disease [13]. Aging is characterized by a progressive decline in cellular functions driven by interconnected hallmarks, among which mitochondrial impairment plays a pivotal role [35]. These interconnected physiological domains are also biologically linked to mitochondrial processes, including cellular energy metabolism, redox homeostasis, inflammatory regulation, and stress adaptation [36]. Therefore, integrative metrics that incorporate multidimensional physiological domains may offer a more comprehensive understanding of physiological status than individual biomarkers [37].

The MPHI was constructed by integrating five physiologically relevant domains, namely metabolic status, inflammatory status, hepato-renal function, psychological well-being, and sleep quality. These domains have each been independently associated with physiological health and processes relevant to apparently healthy aging. Collectively, these domains capture complementary aspects of multidimensional physiological health, including energy metabolism, systemic inflammation, hepato-renal function, psychological well-being, and sleep quality. The MPHI incorporated standardized Z-scores derived from metabolic (HbA1c, uric acid, hemoglobin, HDL-C, LDL-C, triglycerides), inflammatory (CRP, ESR), hepato-renal (AST, SGPT, alkaline phosphatase, gamma-GT, blood urea, serum creatinine), psychological (energy/fatigue, emotional well-being, social functioning, pain, general health, physical health, psychological health, social relationships, environment, wellness scores), and sleep (subjective sleep quality, sleep latency, sleep duration, habitual sleep efficiency, sleep disturbances, daytime dysfunction, global PSQI score) measures. By integrating these multidimensional physiological parameters, the MPHI provides a systems-level measure of multidimensional physiological health across metabolic, inflammatory, hepato-renal, psychological, and sleep-related domains.

Middle-aged males demonstrated elevated Z-scores for HbA1c (0.19) and uric acid (0.77) and showed reduced HDL-C (−0.50), indicative of a higher metabolic and cardiometabolic risk compared with other groups. In contrast, middle-aged females showed lower uric acid Z-scores (−0.26) and higher HDL-C (0.80), consistent with relative metabolic protection in premenopausal females. Triglyceride Z-scores were higher in middle-aged males (0.24) than elderly participants, highlighting age- and sex-dependent differences in lipid metabolism. Inflammatory markers (CRP, ESR) were slightly elevated in middle-aged participants, whereas elderly participants exhibited lower inflammatory Z-scores. This pattern may reflect cohort-specific characteristics, including an apparently healthy-survivor effect, although this cannot be determined from the present cross-sectional study. Hemoglobin Z-scores were higher in middle-aged males (0.65) and lower in middle-aged females (−0.98), consistent with known physiological sex differences in hematology [38]. Z-scores for energy/fatigue and psychological well-being were largely preserved across groups. However, elderly females showed reduced social functioning Z-scores (−0.37), suggesting reduced social engagement, which has been linked to increased vulnerability to mental health issues in older adults. Global PSQI Z-scores suggested slightly poorer sleep in middle-aged males (0.39) and better sleep in elderly females (0.70), indicating that sleep quality does not uniformly decline with age and may be influenced by multiple lifestyle and health-related factors [39].

### 4.3. Multidimensional Health Indexing Reveals Subtle Variation in Physiological Domains Across Age and Sex Groups

The composite domain scores showed numerical variation across the four age- and sex-defined groups across physiological and psychosocial domains. Within this cohort, elderly males and females exhibited relatively more favorable metabolic and hepato-renal domain scores than the middle-aged groups. This pattern may reflect apparently healthy-survivor bias, whereby individuals with more adverse metabolic phenotypes are less likely to survive into older age or participate in studies of apparently healthy aging. However, this interpretation cannot be confirmed in the present cross-sectional study. This interpretation is supported by findings from [40], who demonstrated that individuals with higher fitness in midlife maintain more favorable metabolic profiles over nearly three decades. This aligns with the concept of an apparently healthy-survivor effect, whereby those with adverse metabolic traits such as insulin resistance, obesity, or dyslipidemia are more likely to experience earlier morbidity, leaving a metabolically fitter group represented in older-age cohorts [41].

The two-way ANOVA further clarified these group-level patterns by examining the independent effects of age and sex and their interaction on each composite domain and the overall MPHI. No significant main effect of age was observed for any of the five physiological domains or the MPHI. In contrast, sex had a significant effect on the inflammatory and sleep domains, while a significant age × sex interaction was observed for the sleep domain. This indicates that the observed differences in inflammatory and sleep profiles were more closely associated with sex and, for sleep, with the combined effect of age and sex rather than with age alone. Importantly, the overall MPHI did not show significant effects of age, sex, or their interaction, indicating that the domain-specific differences did not translate into a significant difference in the integrated multidimensional physiological health index.

Numerical differences in the inflammatory domain were observed between male and female groups; however, these differences should be interpreted cautiously given the lack of significant overall multivariate group separation. Compared with males, both middle-aged and elderly women exhibited comparatively lower inflammatory domain scores, suggesting relatively less favorable inflammatory profiles. This observation is consistent with the concept of inflammaging, wherein chronic low-grade inflammation has been reported to disproportionately affect women with advancing age [42]. Our result is also supported by recent evidence that demonstrates that elderly women exhibit higher CRP, ESR, and pro-inflammatory cytokine levels than their male counterparts [43]. Accelerated biological aging, the development of frailty, and cardiometabolic risk have all been linked to this sex-specific inflammatory sensitivity [44].

All groups demonstrated mildly reduced psychological well-being scores, with middle-aged women and elderly men exhibiting the lowest values. Our pattern of findings is consistent with epidemiologic studies reporting sex-specific differences in psychological and affective health among older adults [45,46]. While middle-aged women are more vulnerable to stress-related psychological burden associated with midlife hormonal shifts and caregiving stress [47], elderly males frequently see declines in social involvement and emotional resilience [48].

The sleep domain showed notable numerical variation across the four groups, although these differences do not establish statistically significant sex-specific effects. The observed sleep domain pattern is consistent with a 12-year longitudinal cohort study by [49], which demonstrated that women exhibit higher PSQI scores, shorter sleep duration, and greater longitudinal deterioration in habitual sleep efficiency compared with men. Consistent with these previous observations, our data show that elderly males exhibited more preserved sleep function, whereas elderly females demonstrated marked deficits in sleep quality, strengthening the well-documented susceptibility of aging women to decline in both objective and subjective sleep health [50]. Longitudinal studies have reported greater age-related decline in sleep maintenance among women than men [51], and previous studies have also shown that older women are more likely to experience circadian rhythm disruption, insomnia, and daytime dysfunction [52]. These findings underscore the importance of sleep as a key physiological domain in elderly women, as accumulating evidence shows that fragmented and/or insufficient sleep aggravates systemic inflammation and accelerates physical and cognitive aging aspects [53].

The PCA provided an exploratory visualization of the relationships among the five composite physiological domains rather than evidence of distinct group separation. The first two principal components explained 56.05% of the total variance, with metabolic and hepato-renal domains contributing most strongly to PC1, while psychological, inflammatory, and sleep domains primarily influenced PC2. Although modest differences in group distribution were visually apparent, the extensive overlap of the 95% confidence ellipses indicated substantial similarity in the multidimensional physiological profiles across the four age- and sex-defined groups. Consistent with these observations, neither MANOVA nor PERMANOVA demonstrated statistically significant multivariate differences among the groups, suggesting that most of the observed variability occurred between individuals rather than between age- and sex-defined categories. These findings indicate that, within this apparently healthy cohort, the integrated physiological domains remain broadly conserved despite the subtle domain-specific differences observed in the individual composite scores. This observation is consistent with previous reports showing that apparently healthy aging is characterized by considerable inter-individual heterogeneity, with metabolic, inflammatory, psychological, and sleep-related traits varying more among individuals than across chronological age groups [54].

### 4.4. Multidimensional Physiological Health Index (MPHI) Across Age and Sex

Although the elderly male and female groups displayed numerically higher composite MPHI values compared to the middle-aged groups within this dataset, these differences should not be interpreted as statistically significant age-associated effects or as evidence of superior mitochondrial function in older adults. Aging is generally associated with cumulative changes in cellular and physiological function, including alterations in metabolic resilience, inflammatory regulation, and tissue homeostasis [13]. The modest elevation in MPHI observed here may reflect cohort-specific factors, including the selection of apparently healthy volunteers, relatively favorable physiological profiles among elderly participants, lifestyle characteristics, or differences in metabolic, psychosocial, and sleep-related measures, rather than a generalized age-related physiological advantage. The sensitivity analysis further supported the robustness of the MPHI to reasonable changes in domain weighting. The strong correlations between the original weighting scheme and both the equal-weight model (Pearson’s r = 0.934, *p* < 0.0001) and the perturbed-weight model (Pearson’s r = 0.995, *p* < 0.0001) indicate that the relative ranking of the age–sex groups was largely preserved despite changes in the contribution of individual domains. Thus, the observed group-level pattern was not strongly dependent on the specific biologically informed weighting scheme used to construct the MPHI. Nevertheless, because the weighting scheme was biologically informed rather than empirically derived, independent validation in larger cohorts will be necessary before assigning broader clinical or biological significance to the index. These findings highlight the need to interpret composite indices within the context of their multidimensional inputs and the cross-sectional nature of the present study. The MPHI therefore reflects relative multidimensional physiological status within this study cohort and should not be interpreted as a measure of biological age or mitochondrial function. A limitation of the present study is that direct biomarkers or functional measures of mitochondrial activity, such as ATP production, mitochondrial respiration, mtDNA copy number, reactive oxygen species production, or mitophagy markers, were not assessed. Therefore, future studies should independently validate the MPHI against direct mitochondrial biomarkers and functional measures, as well as relevant clinical outcomes, to determine its biological and construct validity and its potential utility as a multidimensional physiological health index.

### 4.5. Gut Microbiota Stability Across Age and Sex Groups

The gut microbiota is increasingly recognized as a central biological system influencing the trajectory of human aging [55]. Changes in microbial diversity, composition, and metabolic capability across the lifespan have been associated with immunological function inflammation, metabolic balance, and cognitive functions, all important factors in good aging. Previous research indicates that the aging process is associated with restructuring of the gut microbiota and such shifts have been linked to key aging hallmarks such as chronic low-grade inflammation, impaired immune–metabolic regulation and increased susceptibility to age-related diseases [56]. Evidence from long-lived families indicates that while the overall core microbiota remains stable with age, the relative abundances of key taxa, particularly those involved in short-chain fatty acid (SCFA) and amino acid metabolism, shift in ways that may contribute to apparently healthy aging and longevity [55]. Within this context, the present study examined gut microbial diversity and taxonomic structure across middle-aged and elderly participants in our cohort.

Our findings showed highly similar microbial diversity, richness, and community composition across age and sex groups, with no discernible differences at the phylum, class, order, family, genus, or species levels, contrasting with reports of age-related microbial drift in some populations. Alpha and Beta diversity analyses likewise demonstrated overlapping distributions and no clear clustering according to age or sex, indicating a relatively stable gut microbial ecosystem within this apparently healthy cohort. These findings suggest that age-related differences in gut microbial composition may be less apparent in apparently healthy individuals and may vary across study populations, potentially reflecting differences in participant characteristics, lifestyle, diet, environmental exposures or other population-specific factors [10,57].

Beyond the shared species viz., *Agathobacter rectalis*, *Ligilactobacillus ruminis* and *Pediococcus pentosaceus*, our analysis also highlighted several taxa whose presence was restricted to specific age groups. Certain species were uniquely detected in the middle-aged group but absent in the elderly, indicating possible age-associated differences in species occurrence. Conversely, a few species appeared exclusively in the elderly group, indicating modest differences in species composition between the age groups. The detection of group-specific species suggests subtle differences in species-level composition despite the overall similarity of the gut microbial communities. Although modest, these differences are consistent with fine-scale variation in species composition rather than broad alterations in community structure, aligning with the overall microbial stability observed in this apparently healthy cohort.

Specifically, *Bifidobacterium bifidum*, *Dialister hominis*, *Ruminococcus_E sp900314705*, *Gemmiger qucibialis* and *Phocaeicola vulgatus* were prominent only in middle-aged men, whereas *Lactiplantibacillus plantarum* was enriched in elderly men. In middle-aged females, *Megasphaera sp000417505* was among the top species, and in elderly females, *Succinivibrio sp000431835* appeared uniquely. These differences suggest subtle shifts in the species-level composition that do not reflect broad taxonomic drift but rather age- and sex-associated fine-scale variation.

### 4.6. Microbial–Host Physiological Correlation Profiling

Exploratory correlation analysis between gut microbial species and multidimensional host parameters identified nominal age- and sex-associated correlations. However, none of these associations remained statistically significant after Benjamini–Hochberg false discovery rate (FDR) correction. Therefore, the observed correlations should be considered exploratory findings, while potential links to microbial metabolite production, mitochondrial β-oxidation, lipid handling, neurotransmitter pathways, or cytokine signaling represent literature-based biological interpretations rather than mechanisms directly measured in the present study. Detailed group-specific correlation results and their biological interpretation are provided in the Supplementary Materials.

While sensitivity analyses demonstrated that the MPHI was robust to alternative weighting strategies, the proposed weighting scheme was biologically informed rather than empirically derived and requires independent validation against relevant clinical outcomes and other measures of physiological health in larger cohorts. Direct mitochondrial biomarkers were not assessed in the present study; therefore, any relationship between MPHI and mitochondrial function remains to be independently established. The operational definition of health was based on participant self-report and study screening procedures rather than comprehensive clinical diagnostic evaluation or medical record verification. Consequently, the findings should be interpreted within the context of an apparently healthy community-based cohort. Although the present study identified stable taxonomic profiles together with exploratory microbiota–host physiological associations, it did not investigate the functional metabolic capacity of the gut microbiome. Functional profiling approaches based on metagenomic pathway reconstruction, such as KEGG, MetaCyc, or HUMAnN analyses, as well as targeted evaluation of short-chain fatty acid biosynthesis and other microbial metabolic pathways, could provide additional mechanistic insight into whether functional alterations occur despite the observed taxonomic stability. Furthermore, although detailed dietary intake information, including habitual consumption of cereals, millets, fermented foods, dairy products, fruits, vegetables, pulses, and other food groups, was collected from all participants using a structured dietary questionnaire, these data were not incorporated into the present microbiome analyses. Dietary patterns and lifestyle factors can substantially influence gut microbial composition and may contribute to both microbiome stability and the group-specific taxonomic patterns observed in this study, particularly within the heterogeneous dietary and lifestyle context of the Indian population [58]. Information on other potentially important confounders, including physical activity, smoking status, alcohol consumption, socioeconomic status, and pregnancy/lactation status, was also collected through the study questionnaire but was not systematically incorporated into the present microbiome and physiological analyses. Therefore, some of the observed group-specific microbial patterns may reflect differences in diet or lifestyle rather than age or sex alone, and residual confounding cannot be excluded. Medication use and menopausal status were likewise not systematically incorporated into the present analysis. Therefore, residual confounding cannot be excluded, and the observed age- and sex-associated patterns should be interpreted cautiously. In addition, the relatively modest sample size limited external validation of the proposed multidimensional framework and restricted the application of more complex multivariable modeling. Larger, independent, and adequately powered prospective cohorts incorporating comprehensive assessment of dietary intake, physical activity, medication use, smoking status, alcohol consumption, socioeconomic status, and menopausal status will be required to enable more robust adjustment for potential confounding and to confirm the robustness and generalizability of the proposed framework. As habitual diet is a major determinant of gut microbial composition and function, future studies integrating dietary intake with metagenomic functional profiling and multidimensional physiological indices such as the MPHI may further help distinguish diet-associated microbial variation from age and sex-related physiological patterns and provide deeper insight into microbiome–host interactions during apparently healthy aging.

## 5. Conclusions

Our study provides an integrated assessment of multidimensional physiological health and gut microbiota structure in a cohort of apparently healthy middle-aged and elderly adults. By constructing a composite MPHI based on metabolic, inflammatory, hepato-renal, psychological, and sleep domains, we observed numerical variation in multidimensional physiological profiles across age- and sex-defined groups; however, multivariate analyses did not demonstrate statistically significant overall differences among the four groups. Two-way ANOVA further indicated that the observed differences were domain-specific, with significant effects of sex on the inflammatory and sleep domains and a significant age × sex interaction for sleep, while the overall MPHI showed no significant effects of age, sex, or their interaction. Descriptively, middle-aged males showed lower composite scores in some domains, whereas elderly participants showed comparatively more favorable metabolic and inflammatory profiles; these patterns should be considered exploratory given the absence of significant overall multivariate group differences.

Taxonomic stability across age and sex groups was found in the gut microbiota assessment, with no significant variations in community structure, richness, or diversity. These findings suggest that gut microbial composition may be more resistant to chronological aging in apparently healthy adults with relatively stable lifestyle characteristics than is generally believed. However, subtle age- and sex-specific species-level variations were present, indicating fine-scale ecological shifts that may precede broader microbial drift.

Exploratory microbiota–host correlation analyses identified age- and sex-associated patterns of nominal associations across metabolic, inflammatory, psychological, hepato-renal, and sleep domains. Several lactate-utilizing and SCFA-producing taxa showed nominal associations with metabolic, inflammatory, and sleep-related domains, consistent with the previous literature, although these findings should be interpreted as exploratory. Exploratory nominal associations were also observed between certain microbial taxa and hepato-renal and sleep-related parameters; however, these associations did not remain statistically significant after FDR correction. These exploratory findings suggest that microbiome profiling may provide complementary insights into multidimensional physiological variation; however, these observations require validation in larger independent cohorts.

Taken together, our study suggests that integrating multidimensional health parameters with microbiome profiling yields a more nuanced understanding of apparently healthy aging than either system alone. While the observed microbiota–host associations are biologically plausible and broadly consistent with the previous literature, none remained statistically significant after FDR correction and should therefore be regarded as exploratory and hypothesis-generating until validated in larger independent cohorts. The composite MPHI approach captures modest multidimensional physiological variation that may not be evident from individual clinical markers alone. Future studies in larger, longitudinal cohorts are needed to validate the MPHI framework, clarify the relationships between gut microbial species and functional physiological domains, and determine whether microbiome-targeted or lifestyle interventions can support physiological health during apparently healthy aging.

## Figures and Tables

**Table 1:** Clinical, metabolic, inflammatory, and well-being profiles across age and sex groups.

Variable	Reference Range/Units	Middle-Aged (40–59 Years) Male (*n* = 13)	Female (*n* = 11)	Elderly (≥60 Years) Male (*n* = 13)	Female (*n* = 8)	*p*-Value
**Demographics**						
Age (years)	—	47.92 ± 5.42	48.55 ± 6.82	66.46 ± 5.14	67.12 ± 8.06	0.0001
BMI (kg/m^2^)	18.5–24.9	24.86 ± 2.83	25.16 ± 2.92	25.04 ± 2.86	25.27 ± 3.33	0.584
**Hematological Parameter**						
Hemoglobin (g/dL)	M: 13.5–18.0 F: 11.5–16.0	15.05 ± 0.76	12.23 ± 1.60	14.29 ± 2.00	13.80 ± 0.39	0.0034
**Metabolic Markers**						
Fasting Blood Sugar (mg/dL)	<100	88.23 ± 11.29	90.00 ± 5.42	90.77 ± 6.76	92.50 ± 15.63	0.492
HbA1c (%)	≤5.6 Normal	5.55 ± 0.44	5.55 ± 0.38	5.35 ± 0.27	5.47 ± 0.59	0.531
Serum Uric Acid (mg/dL)	2.4–7.0	6.14 ± 1.60	4.56 ± 1.02	4.65 ± 1.43	4.11 ± 1.14	0.014
**Lipid Profile**						
Triglycerides (mg/dL)	<150	173 ± 97.49	164.20 ± 44.67	145.2 ± 59.22	133.56 ± 39.32	-
HDL-C (mg/dL)	M > 55 F > 65	45.80 ± 6.87	51.80 ± 4.61	47.44 ± 5.25	46.74 ± 4.28	-
LDL-C (mg/dL)	<100	96 ± 23.85	100.18 ± 22.61	108.91 ± 29.68	109.87 ± 35.04	-
**Inflammation and Liver Function**						
CRP (mg/L)	≤6	2.96 ± 1.67	3.15 ± 1.65	2.76 ± 1.41	2.62 ± 1.14	-
ESR (mm/h)	M ≤ 20 F ≤ 30	15.8 ± 8.41	31.9 ± 11.21	23.24 ± 12.98	21.48 ± 13.5	-
SGOT (U/L)	M ≤ 32 F ≤ 38	22 ± 6.91	17.8 ± 4.8	20.93 ± 7.29	21.74 ± 8.3	-
SGPT (U/L)	M ≤ 41 F ≤ 31	24.4 ± 15.70	22.2 ± 8.65	23.49 ± 11.93	25.74 ± 14.39	-
**Renal Function**						
Blood Urea (mg/dL)	10–45	28.5 ± 5.44	23.9 ± 5.71	24.38 ± 6.65	25.41 ± 6.07	-
Serum Creatinine (mg/dL)	0.4–1.4	0.83 ± 0.18	0.74 ± 0.12	0.75 ± 0.15	0.76 ± 0.12	-
**Well-Being/Sleep**						
Global PSQI Score	—	7 ± 3.85	6 ± 4.32	5.7 ± 3.96	5.7 ± 3.65	-
WHOQOL Physical Domain	—	80.3 ± 12.0	85.8 ± 10.21	84.11 ± 10.46	83.7 ± 9.69	-
WHOQOL Psychological Domain	—	65.5 ± 17.71	68.9 ± 14.34	68.77 ± 12.1	67.59 ± 11.27	-
WHOQOL Social Functioning	—	97.5 ± 7.90	88.75 ± 20.79	76.14 ± 13.43	72.89 ± 10.77	-
SF36 Energy/Fatigue	—	79.5 ± 14.80	82 ± 13.85	81.93 ± 14.6	81.11 ± 14.3	-

Values are presented as mean ± SD. *p*-values represent overall statistical comparisons across the four age–sex groups. Normally distributed variables were analyzed using one-way ANOVA followed by Tukey’s HSD post hoc test, while non-normally distributed variables were analyzed using the Kruskal–Wallis test followed by Dunn’s post hoc test. Pairwise post hoc comparisons were performed for variables showing a significant overall group difference. A dash (–) indicates that the reference range is not applicable.

**Table 2:** Principal component analysis (PCA) loading matrix for the composite physiological domains.

Composite Physiological Domain	PC1	PC2
Metabolic	0.629	−0.322
Inflammatory	−0.398	−0.497
Hepato-renal	0.555	−0.388
Psychological well-being	0.369	0.546
Sleep	−0.042	−0.449

**Table 3:** Two-way ANOVA of MPHI domain scores by age and sex.

Outcome	Age Effect, *F*(1,41), *p*, η²p	Sex Effect, *F*(1,41), *p*, η²p	Age × Sex, *F*(1,41), *p*, η²p	95% CI, Age Difference	95% CI, Sex Difference
Metabolic	F = 2.16, *p* = 0.149, η²p = 0.050	F = 0.105, *p* = 0.747, η²p = 0.003	F = 1.756, *p* = 0.193, η²p = 0.041	−0.455 to 0.427	−0.588 to 0.266
Inflammatory	F = 0.445, *p* = 0.509, η²p = 0.011	** F = 4.968, *p* = 0.031 *, η²p = 0.108 **	F = 0.308, *p* = 0.582, η²p = 0.007	−0.387 to 0.954	−0.999 to 0.298
Hepato-renal	F = 0.225, *p* = 0.638, η²p = 0.005	F = 1.712, *p* = 0.198, η²p = 0.040	F = 0.002, *p* = 0.963, η²p = 0.000	−0.471 to 0.663	−0.300 to 0.797
Psychological	F = 0.003, *p* = 0.957, η²p = 0.000	F = 0.002, *p* = 0.962, η²p = 0.000	F = 0.019, *p* = 0.892, η²p = 0.000	−1.351 to 1.177	−1.303 to 1.142
Sleep	F = 2.731, *p* = 0.106, η²p = 0.062	** F = 6.600, *p* = 0.014 *, η²p = 0.139 **	** F = 6.438, *p* = 0.015 *, η²p = 0.136 **	−0.847 to 0.336	−1.593 to −0.449
MPHI	F = 2.986, *p* = 0.092, η²p = 0.068	F = 1.417, *p* = 0.241, η²p = 0.033	F = 0.956, *p* = 0.334, η²p = 0.023	−0.229 to 0.331	−0.475 to 0.066

Two-way factorial ANOVA was used to assess the independent effects of age category and sex and their interaction on each composite physiological domain and the MPHI. Partial eta-squared (η^2^p) represents effect size. Ninety-five-percent confidence intervals (CIs) are provided for the estimated mean differences across age groups and across sex groups. * *p* < 0.05, ** *p* < 0.01. *n* = 45; residual degrees of freedom = 41.

**Table 4:** Sensitivity analysis of the multidimensional physiological health index (MPHI) using alternative weighting strategies.

Group	Original Weighting (35/25/20/10/10)	Equal Weighting (20/20/20/20/20)	Perturbed Weighting (30/30/20/10/10)
Middle-Aged Male	−0.0413	−0.0438	−0.0248
Middle-Aged Female	−0.1275	−0.1322	−0.1458
Elderly Male	0.0747	0.0672	0.0808
Elderly Female	−0.0072	−0.0651	−0.0292

**Table 5:** Taxonomic richness across age–sex groups at multiple hierarchical levels.

Group	Phylum	Class	Order	Family	Genus	Species
Middle-Aged Male	14	426	48	97	373	923
Middle-Aged Female	15	428	47	101	425	898
Elderly Male	15	465	48	106	447	987
Elderly Female	13	428	47	103	440	905

## Data Availability

All relevant data supporting the findings of this study are presented within the article, including figures and tables. The datasets generated and/or analyzed during the current study are publicly available in the NCBI Sequence Read Archive (SRA) under BioProject accession number PRJNA1440449 and can be accessed at https://www.ncbi.nlm.nih.gov/bioproject/1440449 (accessed on 3 September 2026).

## Supplementary Materials

The following supporting information can be downloaded at https://www.mdpi.com/article/10.3390/microorganisms14092097/s1. Supplementary Discussion: Detailed Exploratory Microbiota-Host Correlation patterns [59,60,61,62,63,64,65,66,67,68,69,70,71,72,73,74,75,76,77,78,79,80,81,82,83,84,85,86,87,88,89,90,91,92,93,94,95,96,97,98].
