# Supplementary material for: Multidimensional Physiological and Gut Microbiome Profiling Identifies Subtle Physiological Patterns in an Apparently Healthy Aging Indian Cohort: A Cross-Sectional Observational Study
Source: Microorganisms. 2026 Sep 19;14(9):2097. doi: 10.3390/microorganisms14092097 (PMC13609193; doi:10.3390/microorganisms14092097)
Supplement: Supplementary file 1 [file microorganisms-14-02097-s001.zip › microorganisms-4533409-supplementary.pdf]

Supplementary Discussion: Detailed Exploratory Microbiota-Host Correlation patterns

The microbiota-host correlation analysis identified several nominal associations across the age and sex-defined groups. However, none of these associations remained statistically significant after Benjamini-Hochberg FDR correction. Accordingly, the following observations are presented solely as exploratory and hypothesis-generating findings and should not be interpreted as evidence of confirmed biological associations or causal relationships. The biological interpretations discussed below are based on previously reported associations in the literature and were not directly assessed in the present study.

In elderly females, species including *Prevotella copri, Limosilactobacillus, Bifidobacterium angulatum,* and *Eubacterium limnosum* showed nominal associations with HDL and other metabolic markers. These taxa include lactate-utilizing and butyrate-producing microbial lineages that have previously been associated with lipid metabolism, energy balance, and metabolic flexibility [59, 60]. Several of these taxa have been reported to include butyrate-producing members, and butyrate has been implicated in mitochondrial β-oxidation and lipid handling in previous studies [61]. Furthermore, butyrate has also been reported to serve as a mitochondrial substrate in colonocytes and to support energy production and mitochondrial respiration [62]. However, butyrate production, mitochondrial β-oxidation, lipid handling, and mitochondrial respiration were not directly assessed in the present study. These findings provide a potential biological context for the observed microbiota-host associations; however, mitochondrial function was not directly assessed in the present study. Lactate-producing/utilizing bacteria also play a key role in metabolic health in aging. Enrichment in lactate dehydrogenase genes associated with microbial lactate generation has been found in centenarians and long-lived persons, indicating an adaptive metabolic role for lactate in maintaining host and gut energy homeostasis [63]. The comparatively favorable inflammatory domain score observed in elderly females was accompanied by exploratory microbial correlation patterns involving several SCFA-associated taxa. However, these observations should be interpreted cautiously, as none of the correlations remained significant after FDR correction and no microbiome-mediated anti-inflammatory effect can be inferred from the present study. The absence of meaningful associations with systemic inflammatory markers such as CRP and ESR further supports the need for validation in larger cohorts. Beyond inflammation, sleep-related variables, which showed some of the strongest correlations in this group, were linked to taxa such as *Prevotella copri, Limosilactobacillus mucosae,* and *Pediococcus stilesii.* These genera are reported to influence neurotransmitter pathways, tryptophan metabolism and cytokine signalling pathways [64, 65, 66]. Although exploratory, these nominal correlations are biologically plausible because these genera have previously been implicated in neurotransmitter metabolism, tryptophan pathways, and cytokine signalling. Similarly, for host parameters related to hepato-renal activity, the nominal negative correlation between serum creatinine and Mesosutterella multiformis Figuris consistent with previous reports linking *Mesosutterella spp.* to nitrogen metabolism [67] and gut barrier integrity [68], although this relationship requires validation in larger studies. In parallel, the nominal positive association between alkaline phosphatase and the butyrate-producing *Agathobacter rectalis* may reflect subtle microbiota-mediated influences on hepatic or biliary enzyme regulation, aligning with evidence that SCFA-generating taxa can modulate liver-gut axis signaling [69]. Psychological functioning showed a different pattern, lactic-acid–producing bacteria such as *Caproicibacterium, Lactiplantibacillus,* and *Pediococcus* were negatively linked with social functioning, several of these same genera showed positive correlations with the WHO-QOL environment domain, specifically *Caproicibacterium, Limosilactobacillus,* and *Paucilactobacillus*. These associations may reflect relationships between microbial metabolism, immune-neurochemical pathways, and psychosocial measures, although the underlying mechanisms cannot be established from the present study. An abundance of information has reported that the strains of *Lactiplantibacillus plantarum* improve stress and emotional balance [70], and *Pediococcus* strains offer mood regulation [71]. These nominal associations are consistent with previous literature describing potential links between lactate-associated bacteria and psychological or emotional well-being; however, their relevance to the observed psychosocial measures in the present cohort

requires validation in larger studies [59].

In elderly males, the gut-host associations formed a broad and interconnected pattern across various domains. The nominal positive associations of several butyrate-producing and SCFA-associated taxa (*Agathobacter rectalis, Acetatifactor spp., Cryptobacteriods spp.*) with HDL and triglycerides are consistent with this group's moderately positive metabolic index and with previous reports linking these taxa to lipid metabolism and metabolic resilience [72, 73, 74]. Furthermore, the presence of pro-inflammatory microbial signatures (apart from *Blautia)* and the CAG-group taxa negatively correlated with urea and other metabolic stress markers are consistent with the favourable inflammatory index, suggesting a microbiome that could potentially mitigate systemic inflammation. In contrast, the slightly negative hepato-renal score corresponds to the observed positive correlation of SGPT with *Paucilactobacillus vaccinostercus* and the elevated urea associated with *Megamonas*, which may be consistent with previous reports linking these taxa to hepatic and renal physiology [75]. *Bifidobacterium longum* and *Methanobrevibacter*, two genera reportedly linked to circadian rhythm modulation [76] and gut-brain metabolite signalling [77], have positive associations with sleep duration. These nominal associations are consistent with the comparatively favorable sleep index observed in elderly males and with previous reports implicating these taxa in circadian rhythm modulation and gut-brain signalling [78, 79]. The nominal associations between *Prevotella copri* and well-being are broadly consistent with previous reports linking this taxon to host metabolic function. Similarly, the comparatively lower psychological well-being score observed in this group is consistent with the nominal negative associations involving *Methanobrevibacter* and *Ruminococcus_C*, taxa that have previously been linked to stress-related metabolic pathways [48, 80]. In middle-aged female, the exploratory microbiota-host relationships put forward the well-characterized beneficial taxa, especially those involved in SCFA synthesis and glycaemic control, define a metabolic and inflammatory profile. The nominal negative association between *Faecalibacterium prausnitzii* and CRP is consistent with its well-established anti-inflammatory properties, as this species has shown ability to systemic inflammation and improve mucosal immune regulation [81]. The absence of nominal microbial correlations with ESR suggests that inflammatory variation in this cohort may be limited or may not be captured by this marker. Glycemic parameters showed a more complex pattern; *Agathobacter faecis* demonstrated a positive association with FBS, the negative correlations observed for *Blautia_A* and *Collinsella spp.* align with evidence that members of these genera contribute to carbohydrate fermentation and enhanced insulin sensitivity [82]. The nominal positive association between HbA1c and *Faecalibacterium prausnitzii* may reflect compensatory expansion of this beneficial taxon in response to glycemic stress rather than a causal link, a pattern previously described in cohorts undergoing metabolic transition in midlife [83]. Hepatic enzyme patterns also demonstrated targeted microbial involvement, with SGPT positively correlated with *Alloprevotella*, a genus roles previously associated with in amino acid fermentation and hepatic metabolic modulation [84], and Gamma-GT negatively correlated with *Blautia_A* and *Dialister*, two taxa previously associated with reducing oxidative stress and bile acid dysregulation [85, 86]. The observed negative correlations such as those involving *Blautia_A, Dialister hominis,* and *Lactiplantibacillus plantarum* may indicate localized or pathway-specific microbiota-host interactions rather than broad differences in psychological functioning. The nominal positive association between *Faecalibacterium prausnitzii* and social relationship scores is consistent with previous literature linking butyrate-producing bacteria to neuroimmune regulation and psychosocial well-being [87]. Although exploratory, this nominal association is consistent with previous reports implicating *Lactiplantibacillus* species in vagal signalling, tryptophan metabolism, and GABA-related pathways that may influence circadian regulation and sleep maintenance [88].

The exploratory microbiota-host correlation analysis in middle-aged males identified several nominal associations that broadly paralleled the composite physiological domain scores. *Faecalibacterium prausnitzii_G* was positively correlated with FBS. While *Faecalibacterium* species are reported to be beneficial, this subgroup-specific trend may indicate compensatory changes in gut ecology in those

with higher glucose levels [89]. Furthermore, there was a nominal positive correlation between triglycerides and *Ruminococcus_Esp*, genera commonly associated with lipid dysregulation and fermentation of carbohydrates [90]. These nominal associations may partly reflect the variation observed in the metabolic composite domain within this cohort. Several nominal associations were observed between inflammatory markers and SCFA-producing taxa. *Megasphaera elsdenii* and CRP had a negative correlation, whereas *Gemmiger formicilis* and ESR had a negative correlation. Both taxa have previously been reported to produce SCFAs with anti-inflammatory properties [91]. These nominal associations are consistent with the comparatively favorable inflammatory domain observed in this group but should be interpreted cautiously. Within the hepato-renal markers, alkaline phosphatase demonstrated a negative correlation with *Faecalibacterium saccharivorans*, consistent with previous reports linking this species to gut-liver homeostasis [92] Conversely, gamma-GT showed a positive association with *Prevotella copri*. This particular species has previously been associated with dietary patterns and hepatic metabolic pathways [93]. Furthermore, SGPT showed a positive correlation with *Prevotella copri_A* and a negative correlation with *Faecalibacterium prausnitzii_D*. Collectively, these nominal associations are broadly consistent with the comparatively lower hepato-renal composite scores observed in middle-aged males. *Faecalibacterium prausnitzii_G* showed a negative connection with psychological characteristics viz., energy/fatigue ratings [94]. Previous studies have linked *Faecalibacterium prausnitzii* to gut barrier integrity and neuroimmune regulation [95]. The nominal association observed here should therefore be interpreted cautiously and warrants validation in larger cohorts. In addition, pain score is positively correlated with Bifidobacterium longum but negatively correlated with *Faecalibacterium prausnitzii_D* and *Gemmiger formicilis*. These nominal associations are consistent with previous reports linking SCFA-producing bacteria to inflammatory and pain-related pathways, although no causal relationship can be inferred [96]. Several beneficial taxa, including *Blautia*, *Gemmiger formicilis*, and *Ruminococcus_Esp*, demonstrated unfavourable relationships with social functioning scores. These organisms contribute to the synthesis of SCFA and neuroimmune functioning [97]. These exploratory associations are consistent with previous literature describing roles for these taxa in SCFA production and neuroimmune signalling, although their relationship with social functioning remains to be established. Sleep disturbances showed broad microbial involvement. Overall sleep disruption ratings showed a positive correlation with *Acetatifactor intestinalis* and *Streptococcus infantarius*. *Phocaeicola vulgatus* and CAG-group taxa, which are occasionally associated with metabolic stress and circadian disturbance, showed favourable correlations with sleep latency [98]. Although exploratory, these nominal associations are consistent with the comparatively less favorable sleep composite scores observed in middle-aged males and with previous reports linking these taxa to metabolic and circadian pathways.
